# Novel insights on the geomagnetic field in West Africa from a new intensity reference curve (0-2000 AD)

**DOI:** 10.1038/s41598-020-57611-9

**Published:** 2020-01-24

**Authors:** Lisa Kapper, Vincent Serneels, Sanja Panovska, Rafael García Ruíz, Gabrielle Hellio, Lennart de Groot, Avto Goguitchaichvili, Juan Morales, Rubén Cejudo Ruíz

**Affiliations:** 1 National Archeomagnetic Service, Institute of Geophysics, Campus Morelia, Universidad Nacional Autónoma de México (UNAM), Morelia, 58190 Mexico; 2 Department of Geosciences, University of Fribourg, Fribourg, 1700 Switzerland; 3 Deutsches GeoForschungsZentrum GFZ, Helmholtz Zentrum Potsdam, Telegrafenberg, 14473 Potsdam, Germany; 4 Laboratory of Planetology and Geodynamics, Nantes University, Nantes, 44322 France; 5 Paleomagnetic laboratory Fort Hoofddijk, Utrecht University, Utrecht, 3584 CD The Netherlands

**Keywords:** Geomagnetism, Palaeomagnetism

## Abstract

The geomagnetic field variations on the continent of Africa are still largely undeciphered for the past two millennia. In spite of archaeological artefacts being reliable recorders of the ancient geomagnetic field strength, only few data have been reported for this continent so far. Here we use the Thellier-Coe and calibrated pseudo-Thellier methods to recover archaeointensity data from Burkina Faso and Ivory Coast (West Africa) from well-dated archaeological artefacts. By combining our 18 new data with previously published data from West Africa, we construct a reference curve for West Africa for the past 2000 years. To obtain a reliable curve of the archaeointensity variation, we evaluate a penalized smoothing spline fit and a stochastic modelling method, both combined with a bootstrap approach. Both intensity curves agree well, supporting the confidence in our proposed intensity variation during this time span, and small differences arise from the different methodologies of treating data and uncertainties. Two prominent peaks at around 740 AD and 1050 AD appear to be common in ours and several reference curves from other locations, indicating a general westward movement from China to Hawaii of a rather stable feature of the geomagnetic field. However, independent smaller peaks that do not correlate in different locations may hint to localized expressions of the geomagnetic field as a result of temporarily varying non-dipole sources.

## Introduction

Archaeological artefacts have already in the 1930s been proven to be excellent recorders of the geomagnetic field (GMF) for the past 10 millennia^1^. This type of material acquires a thermoremanent magnetization (TRM) at the time of its heating and subsequent cooling that is proportional to the ancient GMF intensity, or archaeointensity. This TRM is dependent on many factors including the ancient heating temperature, the type of magnetic mineral carrying the magnetization, and the reheating process.

Since the beginning of archaeomagnetism, retrieving archaeointensities has been considered a demanding process. Several methods have been developed, for example the thermal Thellier-family methods^2–4^, the microwave technique^5,6^, the multispecimen methods^7,8^ and non-heating methods^9,10^. One of the non-heating methods, the relative pseudo-Thellier technique^11^, makes use of alternating magnetic fields to demagnetize the samples and has recently been successfully calibrated and applied to volcanic rocks^12–14^. However, the calibrated pseudo-Thellier method has never been applied to archaeological artefacts before. The Thellier-family methods are considered as the most reliable of all techniques, because they have been used since nearly 100 years and include many quality assessments. These methods can comprise up to 50 time-consuming heating steps. On the contrary, the pseudo-Thellier method is much faster and avoids chemical alteration of the samples.

Archaeological artefacts have been collected from many locations world wide to construct palaeosecular variation curves, or reference curves, of the GMF intensity. Many regional reference curves have been created for, e.g., West Europe^15,16^, East Europe^17,18^, China^19^, Mesoamerica^20^ and Hawaii^21^. These curves give insights on the variation of the geomagnetic field on a regional, as well as on a global scale. Furthermore, they can be used to date newly found archaeological artefacts in these regions by comparing their magnetic signal to the known reference curves^22^. Up to now, many studies assessed whether certain intensity features, such as periods of strong field strength observed in Europe at around 800 AD^15^, and 200 years later in Hawaii^12^, are connected and arise from the same geomagnetic phenomena that traverse Earth, or whether they occur independently.

Regional reference curves are constructed using data from a circular area with a radius usually of about 700-1200 km^16,17,23^ around the region of interest. The data need to be relocated to a central reference point, which introduces an error^24^ depending on the distance from the relocation point and the geographic area. However, the current data are inhomogeneously distributed in space^25,26^. For example, on the whole continent of Africa and for the last two millennia, the archaeomagnetic Geomagia50.v3^27^ database yield only 46 archaeointensities, and additional publications^28–30^ provide 13 more intensities. This data gap may hinder an investigation of regional GMF phenomenona, or may even introduce a bias in global geomagnetic field models over Africa. For West Africa, a collection of 17 high-quality archaeointensity data from 1000 BC to 1000 AD has been obtained by Mitra *et al*.^31^. They noticed an intensity high around 700 AD in Senegal and Mali and observed a small temporal offset in the data from regions further to the north. This offset is interpreted as evidence for a non-axial dipolar contribution of the GMF in West Africa.

Creating reference curves from data is a notorious challenge and many different techniques have been used in the past. For example, the iteratively re-weighted least-squares fit with a Huber norm used by Thébault *et al*.^32^ allows to mitigate the effect of outliers and to estimate a probability density function (pdf) of a palaeosecular variation curve without strict assumptions on its form. The Bayesian approach introduced by Lanos *et al*.^33,34^ uses variable window widths along the time axis that are adapted to the density of data points. This method takes measurement and dating errors into account, and incorporates physical assumptions of the GMF through prior probability density functions. The stochastic modelling approach introduced by Hellio *et al*.^35^ relies on a time-correlation function chosen to be compatible with present knowledge about the geomagnetic time spectrum. Little attempts have been made to apply different methods on the same data to confirm their consistency^15^.

Here we present new archaeointensities from Burkina Faso and first intensity determinations from Ivory Coast, West Africa. Samples of iron furnace walls were collected from Korsimoro (KRS; Burkina Faso) and Doumbala and Siola (DMB and SIO; Ivory Coast). Their ages were thoroughly determined with radiocarbon dating and from the archaeological context. Samples from Doumbala cover a total age range from 1300–1650 AD, those from Siola from 1000–1900 AD, and those from Korsimoro from 650–1700 AD. We compare the thermal Thellier-Coe^2,36^ with the calibrated pseudo-Thellier method to assess if the latter approach proves suitable for archaeological artefacts. Furthermore, we set up reference curves for West Africa for 0–2000 AD using a penalized smoothing splines technique and a stochastic modelling approach, both using a bootstrap sampling.

## Rock Magnetic and Demagnetization Analysis

To assess the suitability of our samples as stable recorders of the GMF, we subject samples from all sites to a rock magnetic study comprising measurements of thermomagnetic, hysteresis, isothermal remanence and backfield curves. The results are summarized in Suppl. Table S1.

Thermomagnetic curves of samples from DMB can be separated into two groups: samples with one Curie temperature, *T*_*c*_, (Fig. S1a) and samples with two inflections (Fig. S1b). The first inflection is common for both groups. It is on average 535 ± 28°C indicating the *T*_*c*_ of Ti-magnetite. The three samples of the second group have an additional inflection at around 330°C or at 133°C. Curves with two inflections are reversible, while curves from the first group are nearly reversible. Hysteresis curves as well have two distinct shapes: on the one hand we observe narrow (Fig. S2a) and on the other hand, wasp-waisted curves (Fig. S2b). Coercivities (*B*_*c*_) range from 7.0 to 25.8 mT. Samples with wasp-waisted curves have also thermomagnetic curves with two inflections, indicating the presence of a low and a high coercivity mineral. Backfield curves can be separated in two groups: the first group has *B*_*cr*_ < 33 mT (Fig. S3a) and reach saturation in their isothermal remanent magnetization (IRM) curves at 150–200 mT (not shown); while samples from the second group have *B*_*cr*_ > 50 mT (Fig. S3b) and are not saturated at 700 mT in their IRM curves (not shown).

Thermomagnetic and k-T curves of samples from SIO can be divided into two groups: (1) with *T*_*c*_ ~ 571°C (Fig. S1c) indicating magnetite with low Ti content as carrier of the magnetic signal and (2) with *T*_*c*_ ~ 623°C with maghaematized magnetite or Ti-haematite (Fig. S1d). Furnaces that were accepted in the archaeointensity experiments (SIO1, SIO4, SIO5) have also the highest amount of reversible thermomagnetic and k-T curves. Coercivities of the four measured samples are very similar, from 9.2 to 14.5 mT, as well as the shape of their hysteresis curves, supporting maghaematized magnetite rather than Ti-haematite (Fig. S2c). The backfield and IRM curves are saturated around 200 mT (SIO1D and SIO20) or have a very small increase until 700 mT, and *B*_*cr*_ values are very similar with a maximum of 47.7 mT (Fig. S3c).

Of the three measured samples from KRS only KRS34-4C has a completely reversible thermomagnetic curve (Fig. S1e), with a *T*_*c*_ = 559°C indicating Ti-magnetite and a stable magnetization. The other two curves are not reversible with similar *T*_*c*_ = 561°C on average, indicating magnetite with a low Ti content (Fig. S1f). All three hysteresis curves have a relatively high paramagnetic content, compared with curves from DMB and SIO, obvious from the strongly increasing hysteresis arms (Fig. S2d,e). Hysteresis curves have shapes from normal (Fig. S2d) to wasp-waisted (Fig. S2e). Backfield curves reveal larger *B*_*cr*_ than other samples: KRS35-5D has the highest *B*_*cr*_ = 291.0 mT, while the other two have 65 mT on average (Fig. S3d,e). All three samples have unsaturated IRM curves, indicating a contribution of a highly coercive magnetic mineral such as haematite, with KRS35-5D having the largest contribution, supported by hysteresis curves and its high *B*_*cr*_.

Alternating field (AF) demagnetization behaviour of DMB and SIO20 test specimens reveal a characteristic remanent magnetization (ChRM) and a small viscous component that was removed by fields of 7.7 mT on average (not shown). Line fits to isolate the ChRM have maximum angular deviations, MAD < (3.0±1.5)° and deviation angles, DANG < (1.7±1.7)°. These characteristics indicate that the specimens are suitable for archaeointensity experiments. Only four specimens have larger MAD and/or DANG, most probably because they broke during the procedure. Two DMB specimens from DMB5 had more than 60% left of their magnetization after the 80 mT demagnetization step, indicating a high coercive mineral contribution such as from haematite. This is confirmed by the wasp-waisted hysteresis of a specimen from the same furnace (Fig. S2b). Additional demagnetization experiments are ongoing, but were not the focus of this article. The demagnetization behaviour of KRS specimens was comprehensively investigated in Donadini *et al*.^37^. In general, the specimens have a single magnetic component with a weak viscous overprint that is isolated by fields of 5 mT or temperatures of 100°C on average.

In summary, the samples from all three sites appear to be stable recorders of the GMF, with Ti-magnetite as main carrier of the magnetic signal and reversible and nearly reversible curve. Demagnetization reveals a single component of magnetization with a small viscous overprint. Hence, we consider the samples suitable for an archeaointensity study.

## Archaeointensity Results

### Results of the Thellier-Coe (TH-C) experiment

**Figure 1:**
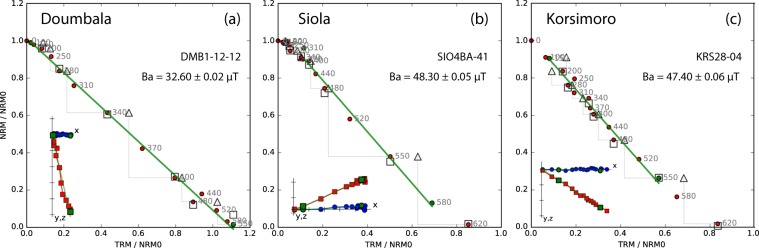
Successful examples of the Thellier-Coe experiment from (**a**) Doumbala, (**b**) Siola and (**c**) Korsimoro. ‘Ba’ is the archaeointensity, the red dots are measurements at different temperatures steps, empty triangles and squares are pTRM- and tail-checks, respectively, the green bold line is the best-fit line, and the inset is the vector diagram with declination (blue circles) and inclination (red squares).

The Thellier-Coe method consists of a step without and a step with an applied magnetic field at each temperature. Temperature steps range from room temperature to 620°C. At each second temperature, pTRM- and tail-checks are performed. In order to exclude unreliable archaeointensities, we accepted specimens based on the following criteria, adapted from Shaar and Tauxe^38^: number of points used for the best-fit slope, *N* ≥ 5; the fraction of remanence used for the best-fit slope, *FRAC* ≥ 0.6; the ratio of the standard error of the slope to the absolute value of the slope, *β* ≤ 0.08; the maximum gap measured in vector-length, *GAP* − *MAX* ≤ 0.6; and the statistics of the directions, the maximum angular deviation, *MAD* ≤ 5°, and the deviation of the angle to ensure that the characteristic component was selected, *DANG* ≤ 5°. We accepted 40 of 54 specimens (success rate of 74%; Table S2). For the best-fit slope we used on average the temperature range 200–500°C comprising nine data points. Arai^39^ diagrams of DMB specimens provide reliable results: all are linear up to at least 400°C (Fig. 1a); one specimen broke during the experiment. Siola specimens provide less reliable Arai diagrams. Besides linear diagrams (Fig. 1b), we observe convex shapes and scattered lines. This scatter also appears in the directional vector diagrams, which we attribute to loose material that was not in place in the rather porous samples. More Arai diagrams of KRS specimens were accepted than of DMB. Besides linear diagrams (Fig. 1c), we observe two slopes and slightly scattered examples. Several KRS specimens have deviating pTRM-checks at 550°C, indicating that chemical alteration has occurred. However, this step was performed after heating to 620°C, at which this type of alteration often happens.

Cooling rate (CR) corrections are nowadays standard in archaeointensity experiments. With this correction the difference in the ancient and the laboratory CR are taken into account. The rapid cooling in the laboratory usually leads to an overestimation of the archaeointensity value^40,41^. We accepted and applied CR corrections for 34 of the 54 specimens. The remaining 20 specimens experienced chemical alteration during the CR experiment. For these specimens we calculated averages of sister specimens, assuming a homogeneous CR within a sample.

To account for a possible influence of the magnetic anisotropy on the determination of archaeointensity, specimens were placed in six directions (−x, +x, −y, +y, −z, +z) in their sample holders for the archaeointensity experiment. We found a low standard deviation, in general, with only one average having *σ*_*Ba*_ > 5 *μ*T and >10% (SIO1; Table 1, S5). The remaining six sample averages have a maximum standard deviation of 4.1 *μ*T, which is similar compared to other determinations^29,31^ (Table 1). The low standard deviations indicate a low anisotropy effect, because the anisotropy correction is not only affected by characteristics of the magnetic grains, but also by the orientation of the NRM respectively to the applied laboratory field (e.g., Veitch *et al*.^42^). Anisotropy corrections of specimens from KRS obtained by Kapper *et al*.^43^ as well are low for the majority of the specimens.

**Table 1:** Furnace averages of archaeointensities with ‘Lat’, the latitude, and ‘Lon’ the longitude. *Age* and *σ*_*Age*_ are the age and its error, respectively. ‘Experiment’ indicates ‘TH-C’ for the Thellier-Coe protocol for specimens from this study, ‘TH-I’, the Thellier-IZZI protocol for reanalysed specimens from Korsimoro^43^ applying the new selection criteria, and ‘P-TH’, for the pseudo-Thellier method from this study. *N*_*meas*_/*N*_*acc*_ indicates the ratio between specimens measured and accepted. Furnace averages were obtained after averaging intensities of first, specimens and then samples. ‘Ba’ are the cooling rate corrected archaeointensities in the case of TH-C and TH-I, and *σ*_*Ba*_ is its corresponding standard deviation.

Furnace	Lat (°)	Lon (°)	*Age* ± *σ*_*Age*_ (yrs AD)	Experiment	*N*_*meas*_/*N*_*acc*_	Ba (*μ*T)	*σ*_*Ba*_ (*μ*T/%)
KRS33	12.81	−0.99	720 ± 60	TH-I	3/3	65.4	9.3/14.3
KRS05	12.81	−1.06	800 ± 90	TH-I	16/6	50.9	1.1/2.3
KRS06	12.79	−1.09	800 ± 90	TH-I	3/3	45.3	9.4/20.7
KRS35	12.81	−1.06	1100 ± 75	TH-I	13/8	32.9	0.4/1.3
KRS35	12.81	−1.06	1100 ± 75	P-TH	5/2	22.3	2.5/11.0
KRS24	12.79	−1.09	1120 ± 90	TH-I	9/2	42.7	0.2/0.5
KRS34	12.81	−1.06	1290 ± 20	TH-I	12/6	38.3	1.1/2.8
KRS10	12.79	−1.09	1340 ± 55	TH-I	8/5	30.5	2.8/9.2
DMB6	9.88	−7.41	1350 ± 65	P-TH	5/3	38.2	1.6/4.2
KRS21	12.79	−1.09	1400 ± 100	P-TH	4/2	26.1	4.9/18.9
KRS23	12.79	−1.09	1430 ± 20	TH-I	12/8	38.2	5.2/13.6
KRS23	12.79	−1.09	1430 ± 20	P-TH	7/6	32.3	2.3/7.2
SIO4	9.86	−7.45	1545 ± 55	TH-C	6/6	47.1	4.1/8.7
DMB3	9.88	−7.41	1550 ± 100	TH-C	6/6	36.6	0.9/2.5
KRS28	12.79	−1.09	1650 ± 50	TH-C	6/6	47.9	3.2/6.7
KRS13	12.81	−1.06	1650 ± 150	TH-I	3/2	51.0	0.2/0.4
KRS30	12.79	−1.09	1650 ± 50	TH-I	3/2	41.3	3.1/7.5
KRS30	12.79	−1.09	1650 ± 50	P-TH	6/3	35.5	13.6/38.3
SIO1	9.86	−7.45	1815 ± 135	TH-C	6/3	41.2	5.4/13.2
SIO1	9.86	−7.45	1815 ± 135	P-TH	5/2	27.7	0.8/3.0
SIO5	9.86	−7.45	1870 ± 60	TH-C	12/8	43.6	1.9/4.3
DMB1	9.88	−7.41	1895 ± 80	TH-C	6/5	30.8	1.4/4.6
DMB2	9.88	−7.41	1895 ± 80	TH-C	6/6	31.3	0.3/0.8
DMB1	9.88	−7.41	1895 ± 80	P-TH	5/4	32.7	3.7/11.4
DMB2	9.88	−7.41	1895 ± 80	P-TH	5/4	31.4	2.1/6.8

### Results of the calibrated pseudo-Thellier (P-TH) experiment

The success rate of the P-TH method on specimen level is rather low compared to the rate of the TH-C method: 46% of DMB specimens, 17% of SIO specimens and 29% of KRS specimens fulfilled the grain size criterion 23 ≤ *B*_1/2*ARM*_ ≤ 63  mT (Table 1, S3, S5). The segment that was chosen to obtain the P-TH slope, was on average 15 mT to 80 mT. On furnace level we obtained 13 average values, of which five were rejected because they contain only one specimen. The three accepted specimens from the Zurich experiment are KRS10-04-H, KRS23-04-B and KRS30-08-M (Table S5). The KRS10 is the only accepted specimen from this furnace. KRS23-04-B agrees with other sister specimens measured in Utrecht, and KRS30-08-M is much higher than its sister specimens causing a large standard deviation. From these results, we do not see a difference between the experiments in Zurich and in Utrecht.

**Figure 2:**
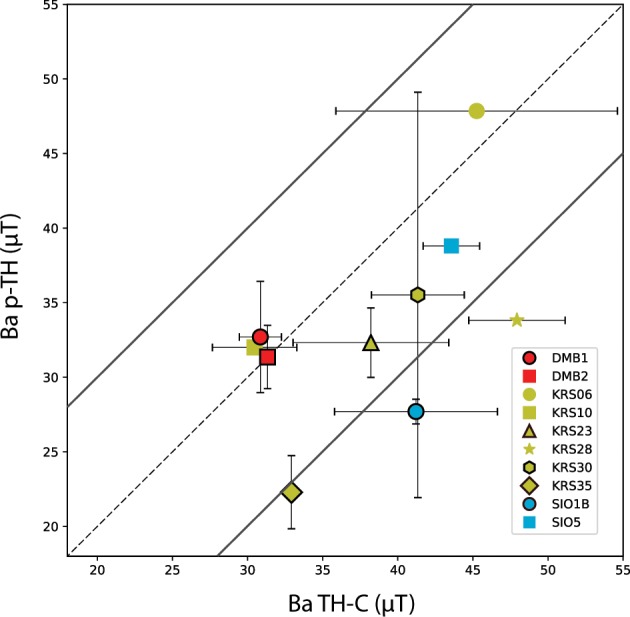
Comparison of pseudo-Thellier (P-TH) and their corresponding Thellier-Coe (TH-C) archaeointensities. Site averages that coincide very well are located close to the dashed line. Continuous lines indicate the error envelope of ±10 *μ*T. Site averages of KRS06, KRS10, KRS28 and SIO5 were obtained from only one specimen in the P-TH experiment and were therefore rejected for the set up of the reference curve (symbols without black outline).

In order to probe the agreement of archaeointensities from the two methods, we compare the six furnace averages obtained with both methods (Fig. 2, Table 2). Method averages of KRS35 and SIO1 have *σ*_*Ba*_ > 5* μ*T and >10%. However, they fall with their error bounds within a ±10* μ*T range (Fig. 2). The p-TH measurement of KRS30 has a large standard deviation (Table 1); however, the method averages agree well (Fig. 2, Table 2). Therefore, we do not reject this furnace average. In Fig. 2 we also include results acquired from one specimen. We notice that the results of DMB2, KRS06, KRS10 and SIO5 agree well for both methods, which supports that these furnaces could be promising for future P-TH experiments.

**Table 2:** Method averages, Av_*meth*_, of (cooling rate corrected) Thellier-Thellier (TT) and pseudo-Thellier (PT) intensity furnace averages, Av_*furn*_. *N*_*spec*_ is the number of specimens used for the average, Age and *σ*_*Age*_ are the age of the furnace and its age uncertainty, *σ*_*meth*_ are the standard error of the method average in *μ*T and in %.

Furnace	*N*_*spec*_	Age (yrs AD)	*σ*_*Age*_ (yrs)	Av_*furn*_ (*μ*T)	Av_*meth*_ (*μ*T)	*σ*_*meth*_ (*μ*T/%)	Method
DMB1	5	1895	80	30.8			TT
DMB1	4	1895	80	32.7	31.8	1.3/4.1	PT
DMB2	6	1895	80	31.3			TT
DMB2	4	1895	80	31.4	31.3	0.0/0.1	PT
KRS23	8	1430	20	38.2			TT
KRS23	6	1430	20	32.3	35.3	4.2/11.8	PT
KRS35	8	1100	75	32.9			TT
KRS35	2	1100	75	22.3	27.6	7.5/27.2	PT
SIO1	3	1815	135	41.2			TT
SIO1	2	1815	135	27.7	34.5	9.6/27.7	PT
KRS30	2	1650	50	41.3			TT
KRS30	3	1650	50	35.5	35.5	4.1/11.6	PT

The low success rate of 29% for all sites combined of the pseudo-Thellier method may be caused by the presence of a hard magnetic phase, which is not eliminated by a 300 mT alternating magnetic field. We did not apply an anisotropy correction on these specimens based on previous low determinations^43^. In total, we obtained ten new data with the TH-C protocol, eight new data with the P-TH protocol, and seven reanalysed data from the TH-I method.

## Palaeosecular Variation Curves and Their Analysis

**Figure 3:**
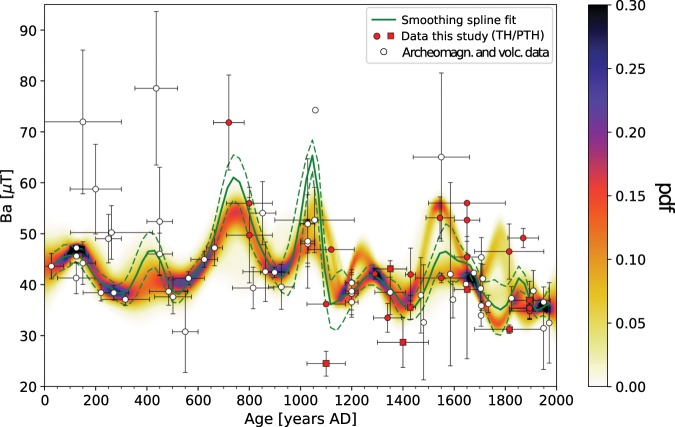
Reference curves for West Africa obtained with the smoothing spline fit (SSF; green, with its 95% error envelope) and with the stochastic modelling approach (SMC; shown as probability distribution function - pdf). White dots are archaeomagnetic and volcanic data from the Geomagia50.v3^27^ database and data not included in the database (please refer to the text for references). Red dots are data from this study obtained with the Thellier-Coe protocol and reanalysed data from Korsimoro from Kapper *et al*.^43^. Red squares were obtained with the pseudo-Thellier method. All data are relocated to the central reference point.

To produce palaeosecular variation (PSV) curves for NW Africa, we combine our newly obtained and reanalysed data with published data from sites within a circle of 2100 km radius around 21°N and 0°E. We adopt two different methods to determine PSV curves: a smoothing spline fit and a stochastic approach, which are described in the ‘Setting up the reference curves’ section. The smoothing spline fit (SSF) and the stochastic modelling curve (SMC) are in general very similar, although the two methods are distinct (Fig. 3). Our data are fitted well by the two approaches. Only KRS35 (P-TH, Table 1) at 1100 AD is lower than other data from the same period and not within the error bounds of the curves. Although this data point is of high quality, the SMC does not take it into account, because it is treated as outlier. On the contrary, this data point slightly affects the SSF by pulling down the error envelope of the curve. The SSF is clearly more sensitive to single high-quality data that lie in greater distance of other data, also visible for a high-intensity data point at 1058 AD. This is probably linked to the use of the L2 norm for the SSF that is more sensitive to outliers than the Huber norm used for the SMC. Two other of our data points are not within the error range of the curves, but they are in close vicinity (KRS28, SIO5, both TH-C; Table 1).

The two curves agree well in the periods 0–1050 AD, 1200–1500 AD, and at around 1650 AD. At 1200–1300 AD and 1760 AD the SMC splits up, while the SSF confirms the younger and the lower peak, respectively. The two curves clearly reflect large uncertainties in the period 1500–1650 AD. Furthermore, in the period from 1500 AD onwards the difference in modelling of the two methods is evident. The use of prior information of the GMF in the SMC method as well as the probability distributions of values per year provide a more precise description of the palaeosecular variation.

**Figure 4:**
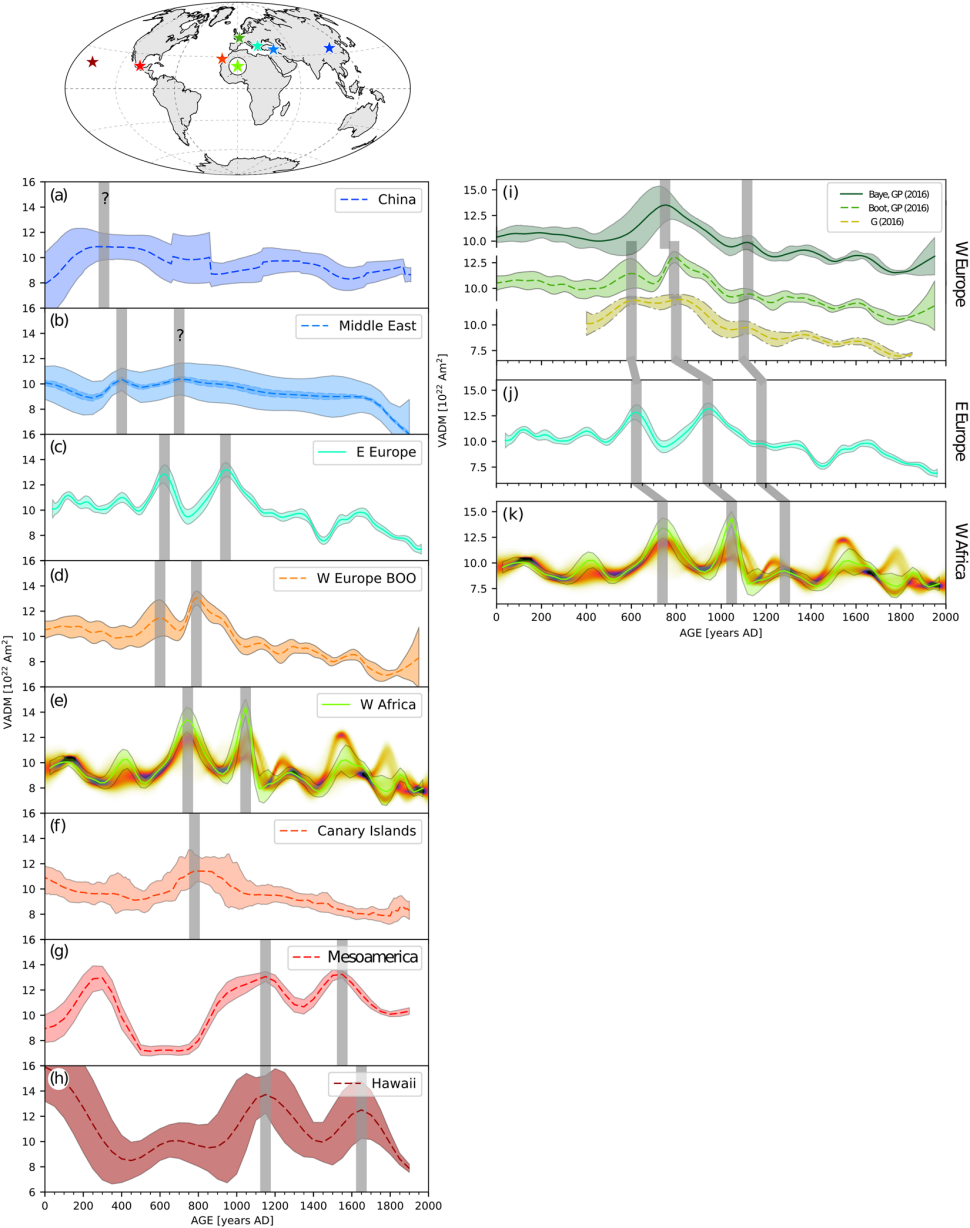
Reference curves from different regions. (**a**) China, (**b**) the Middle East, (**c**) East Europe, (**d**) West Europe (bootstrap curve), (**e**) West Africa from this study, (**f**) Canary Islands, (**g**) Mesoamerica, (**h**) Hawaii. For references please refer to the text. Central location points of the curves are marked in the map above. (**i**–**k**) Comparison of reference curves from West and East Europe, with West Africa. (**i**) the bayesian and bootstrap curves from Gómez-Paccard *et al*.^15^, (**j**) the curve from Genevey *et al*.^16^ and (**k**) the smoothing spline fit obtained from East European data selected from the Geomagia30.v3 database^27^. The strongest peaks are indicated with grey bars. The map in this figure was produced with the Python 2.7.10 software (https://www.enthought.com).

We observe five peaks that coincide in the two curves: at 100 AD, 740 AD, 1050 AD, 1280 AD, and 1550 AD, of which three have higher amplitudes in the SSF. One of the largest peaks in the SMC with high probability is at 740 AD and is confined by three data from this study, data from Mitra *et al*.^31^, Gomez-Paccard *et al*.^44^ and Casas *et al*.^45^. Mitra *et al*.^31^ observed a gradient in intensity between sites from Egypt at higher latitudes and Mali and Senegal from lower latitude, with higher intensities from Egypt. While their highest intensities rise to 47 *μ*T in Senegal and Mali shortly before 700 AD, the intensity peak in the SSF rises much higher, to about 60 *μ*T at 740 AD for the same latitudinal band (Fig. 3). By comparing the data used for the reference curves from northern latitudes from the circular area with those from more southern latitudes we do not observe differences in VADM strength between 750–2000 AD (Fig. S5). Nevertheless, VADMs from Northern latitudes earlier than 750 AD seem to be stronger than those from southern latitudes. However, these data have large uncertainties compared to the more south located data, and the data density is low. Therefore, we tend to a cautious interpretation of the data related to a latitudinal gradient. Furthermore, Gomez-Paccard *et al*.^15^ pointed out that the most significant feature of the GMF in the past 2000 years is an intensity peak at 800 AD in Europe, about 50 years later than in W Africa (Fig. 4).

In two separate sets of analyses (multispecimen and Thellier-Coe analyses), Tarduno *et al*.^29^ observed a sharp drop in archeointensity in the Limpopo Valley region of South Africa starting at 1250 AD that reached its minimum at around 1370 AD. These changes were related to recurrent reversed core flux patches that produced field behaviour similar to that seen in the South Atlantic Anomaly region today. Compared to our curves we note an onset of a field decrease earlier, at 1050 AD, reaching its minimum intensity at about the same time, at 1400 AD (Fig. 3, Table 1). However, we remark that two of our data points drop to much lower intensities already at 1100 AD. Further data collection could help better elucidate trends in the West African curves and allow testing of flux expulsion models and their possible relationship to the South Atlantic Anomaly (e.g., Tarduno *et al*.^29^).

In the period of 1400–2000 AD our data compliment the existing data. For example, at 1550 AD our data point to a rather high field intensity supporting a high intensity from the Canary Islands^46^.

**Figure 5:**
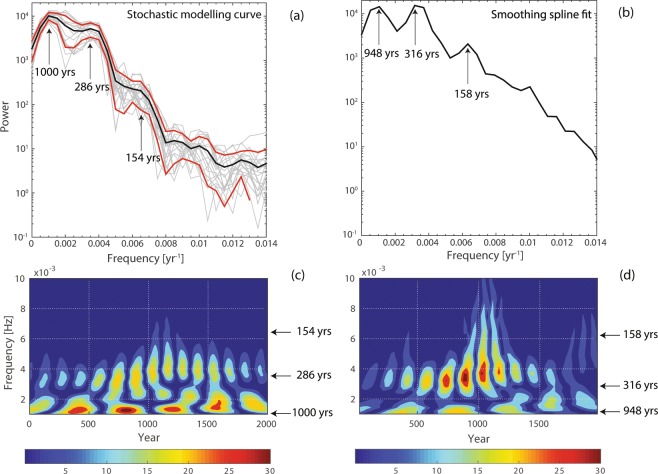
(**a**,**b**) Multitaper power spectra of the WAFR curves. In (**a**) the light grey curves are spectra of each member of the ensemble, while the dark black and red lines are the average curve and the standard deviation, respectively. (**c**,**d**) Morlet wavelet power spectra for the WAFR curves. The wavelet power spectrum is given as a function of frequency and the identified periods are noted on the right. The colour scale denotes the absolute values of the wavelet coefficients.

In order to determine periodicities in the curves we selected the multitaper analysis. The multitaper analysis applied on the SMC reveals maxima in the power spectrum of the average curve at around 1000 years, 286 years and at 154 years. The same analysis performed on the SSF shows very similar periodicities with maxima in the spectrum at 948 years, 316 years and 158 years, confirming the agreement of the two WAFR curves (Fig. 5a,b). The wavelet analysis was then further applied to investigate if these periods persist within the whole period of 2000 years. The analysis of the two curves show that the periods of 286 and 316 years, respectively, are present throughout almost the whole 2000 years but weaken towards both ends of the curve (Fig. 5c,d). Furthermore, we clearly observe the longer periodicity of about 1000 years in both spectra, although it is stronger in the SMC. The shortest period of around 154 years is barely visible in wavelet analysis of the SMC. In conclusion, the wavelet analysis confirms the existence and the stationary nature of a periodicity of about 300 years, which is about 50 years longer than the one observed in a West European curve^16^ between 400–2000 AD. Genevey *et al*.^16^ suggested that the recurrence of peaks in W European might be related to equatorial flux features that have a periodicity of ~250 years at the equator or waves that occur when the top of the liquid core is stably stratified^47,48^.

## Comparison With Other Reference Curves and Global Geomagnetic Field Models

We compare our curves with reference curves from: Hawaii^21^, Mesoamerica^20^, the Canary Islands^46^, West Europe^15^, East Europe - with data from the Geomagia50.v3^27^ from an area of a circle of 700 km radius around Thessaloniki (40.6°, 23.0°) by calculating the curve in the same manner as the SSF, the Middle East^49^, and China^19^ (Fig. 4). The most prominent intensity peaks were marked for each curve. The curves from the Canary Islands and W Africa feature a peak at about the same time period, between 740–800 AD (Fig. 4c,d). These two curves, however, were constructed partly with the same data from W Africa and the Canary Islands. The curve of the Canary Islands has only one, rather broad, peak that may not resolve the two features. Furthermore, we note a peak in intensity around 790 AD also in the curve from W Europe, and a possible peak a bit earlier in the curve from the Middle East (Fig. 4e,f, respectively). The curves from W Europe, W Africa and E Europe have two strong peaks around the same span (600–1100 AD), although they do not coincide. More specifically, the earlier peak appears about 150 years earlier in the two curves from higher latitudes (W and E Europe) than in the more southerly located W African curves (Fig. 4). This feature may evidence some local differences of the GMF, or a movement from North to South. The second explanation seems to hold because the two peaks appear to have a correlated movement.

Comparing the two strongest intensity peaks, we note that they seem to move westward from China to the Middle East, E Europe and to W Africa within 500 years (from 250–750 AD). As mentioned earlier, these two peaks occur at about the same time in W and E Europe, with some differences, and then seem to appear at around 1150 AD and 1550 AD in the Mesoamerican curve and 100 years later in the Hawaiian curve. Assuming a very general movement of the features of 260.5° in 850 years, would indicate a velocity of 0.31°/year.

**Figure 6:**
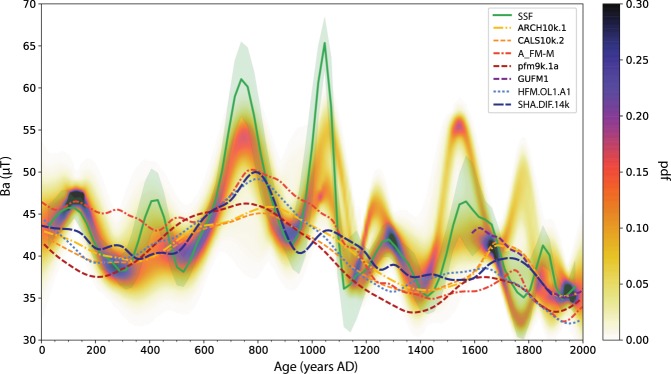
Comparison of West African curves - smoothing spline fit (SSF) and stochastic modelling curve as probability density function (pdf) - with the following global geomagnetic field models: ARCH10k.1^50^, CALS10k.2^50^, A_FM-M^51^, pfm9k.1a^52^, GUFM1^53^, HFM.OL1.A1^50^, SHA.DIF.14k.^26^.

Additionally we compare our curves with several global GMF models: ARCH10k.1^50^, CALS10k.2^50^, A_FM-M^51^ (mean model), pfm9k.1a^52^, GUFM1^53^, HFM.OL1.A1^50^, and SHA.DIF.14k^26^ (Fig. 6). These models cover different periods, from the historical period of 400 years (GUFM1) to the past 14 kyr (SHA.DIF.14k). They are based on volcanic and archaeomagnetic data only (ARCH10k.1, A_FM-M, SHA.DIF.14k), and others include sediment data as well (CALS10k.2, pfm9k.1a, HFM.OL1.A1). The SHA.DIF.14k agrees well with our intensity curve - it exhibits two distinct peaks at 780 AD and 1100 AD and one at 1300 AD with a smaller amplitude. The A_FM-M has similar intensity values between 100-150 AD, but it does not decrease as our curves and the other models later on. Some of the models have lower intensities than our curves over several periods, e.g., between 1150-1400 AD.

## Conclusions

The calibrated pseudo-Thellier method has been applied for the first time on archaeological material. However, the success rate of 29% was low compared to the rate of the Thellier-Coe method (74%). We found that the samples from Doumbala, presenting one stable and ideal magnetic phase, yielded consistent results with both methods. Therefore, we consider the calibrated pseudo-Thellier method as promising for archaeological artefacts, provided that an ideal magnetic phase dominates the magnetic signal. A profound comparison of the calibrated pseudo-Thellier method applied to archaeological artefacts with other archaeointensity methods can be an interesting topic for an additional methodology focused article, but is here out of scope.

The two methods that we chose to construct the geomagnetic field intensity reference curves for West Africa - a smoothing spline fit and a stochastic modelling approach - gave similar results. The stochastic modelling approach has the option to identify periods of high probability, and therefore, of higher confidence. Both methods use different measures of misfit and therefore their sensitivity to data with low/high uncertainty differs. In some cases, the data scatter and intensity uncertainties are large. Nevertheless, our intensity curves from these modelling approaches agree well between 0–1500 AD, providing confidence that these represent the best view of field variation given the available data. The periodicity analysis identifies periodicities of about 300 and 1000 years, of which the former is 50 years longer than a periodicity observed in W Europe. This difference to W Europe may indicate local, non-dipolar effects of the geomagnetic field.

By focusing on the two most prominent peaks at 740 AD and 1050 AD we could track an apparent east- to westward movement of these features. In a broad sense, the largest peaks seem to move westward from China to Hawaii, covering this distance of about 260.5 degrees in longitude in 850 years. This would imply a westward motion of 0.31 degrees/yr. However, the central relocation points of the curves do not lie on the same latitude. By investigating curves from W and E Europe and W Africa, we observe that the same features appear either later in southern latitudes or that they moved southwards.

Differences during several periods between the W African curves and global geomagnetic field models calculated at the reference point, indicate an influence of the abundance of European data and the lack of African data in the global models.

## Samples and Methods

### Archaeological background

**Figure 7:**
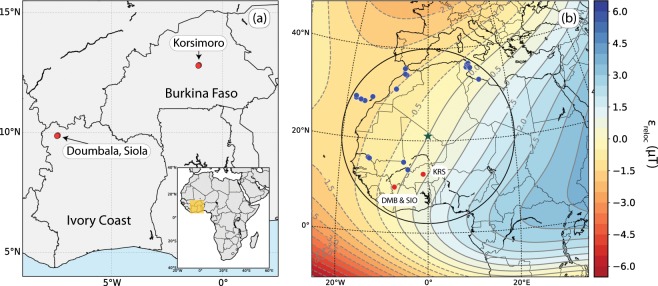
(**a**) Sampling locations in Korsimoro (KRS, Burkina Faso) and Doumbala and Siola (DMB and SIO, respectively, Ivory Coast; red dots). The rectangle in the inset marks the area presented. (**b**) Map showing the relocation point of the data (star), the locations of all data used for the reference curves (blue dots), the radius of about 2100 km within which data was chosen. Contours indicate the relocation error calculated from the IGRF11 model^74^ for 2000 AD. The maps were produced with the Python 2.7.10 software (https://www.enthought.com).

Iron production was essential in the pre-colonial society of West Africa. Iron was used to produce agricultural tools and weapons. Two of the three investigated sites, Doumbala (lat 9.88°, lon −7.41°) and Siola (lat 9.86°, lon −7.45°), are located in NW Ivory Coast, while the third site, Korsimoro (lat 12.79°, lon −1.08°) is located in Burkina Faso 70 km North of the capital, Ouagadougou (Fig. 7).

#### Siola (Ivory Coast)

The village of Siola is located about 6o km North of Odiénne, in the prefecture of Kaniasso and is described in detail in Serneels *et al*.^54^ (Fig. 7a). Siola is located on the right bank of the River Baoulé. Nine areas with notable concentrations of metallurgical sites were found around Siola, of which zones 1000 and 2000 were investigated in detail in the field campaign in January 2012. More than 100 furnace bases and slag heaps were found in these two zones. Nine ^14^C dating revealed a use of the sites during the period 1000–1900 AD. Based on the type of furnace, the amount of slag and the spatial organization of the furnaces, three techniques were identified, namely KAN1, KAN2 and KAN3. KAN1 was used to produce iron during 1300–1450 AD, followed by KAN2 between 1450–1700 AD, and finally KAN3 between 1700–1900 AD. Diameters of the furnaces range from 95 cm (KAN1), to 90–120 cm (KAN2) and up to 100–150 cm (KAN3). Samples were taken from Zone 1000, located north of the village, and from Zone 2000, on the eastern border of Siola.

#### Doumbala (Ivory Coast)

Two zones with remnants close to the village Doumbala were excavated in January 2015 and described in Serneels *et al*.^55^ (Fig. 7a). Doumbala is located 5 km north-east of Siola. One zone forms a band of 100 m width forming an arc of about 600 m length around the village. In this band 49 remnants of furnaces were found. These furnaces reveal similarities to technique KAN3 used in Siola, but the architecture of the furnaces is different. The second zone, Kokoroni, is located 300 m south of the village, is 80 m wide and 400 m long. In Kokoroni 45 bases of furnaces were found. They are related to techniques KAN1 and KAN2 of Siola. Eight ^14^C determination and the archaeological context defined a time range of smelting activities between 1300–1650 AD. Diameters of the furnaces have a range between 90–150 cm. Samples were taken in both zones.

#### Korsimoro (Burkina Faso)

Korsimoro has been thoroughly investigated by V. Serneels in two field campaigns in 2011 and 2012 (Fig. 7a). Archaeological research has revealed 12 zones of metallurgical activity, which were divided into 303 sectors. The zones are distributed over an area of 10 × 6 km along the river Kiétébala, and each sector spans up to 1 km. Four different smelting techniques, corresponding to four subsequent periods, have been identified. The types of furnaces were distinguished based on the grouping and typology of the furnaces, the amount and type of slag deposits, the tuyeres, and their chronology. In total, 19 radiocarbon age determinations were obtained from charcoal and burnt straw. Samples for dating were collected from the bottom of the excavated furnaces or at the basis of the slag deposits. The archaeological age was combined with the ^14^C datings to obtain four better confined, subsequent time intervals. These intervals correspond to the four techniques, which cover a total time period of 650-1700 AD. For more details on the structures and techniques please refer to Serneels *et al*.^56–58^.

## Sampling and Measurements

### Sampling and sample preparation

From each furnace several oriented blocks were collected. The orientation of the in situ samples was registered on the flat surface of a cap of plaster of Paris by marking the sun’s shadow. Samples from Korsimoro were taken during the field campaign in 2012 by F. Donadini. Samples from Siola and Doumbala were taken in 2012 and 2015, respectively. In total, we obtained specimens from six furnaces from Doumbala, from seven furnaces from Korsimoro and from four furnaces from Siola. Hand-blocks were collected homogeneously around the furnace wall. For the pseudo-Thellier experiment cubes of up to 2.2 cm side lengths were used, while for the Thellier-Coe experiment, the cubes were cut in eight subspecimens of 0.5 cm^3^. Six of these were encapsulated in non-magnetic salt pellets and were oriented along the principal axes +x, −x, +y, −y, +z, −z, relative to a chosen x-direction. Morales *et al*.^59^ suggested this protocol of orientations to minimize eventual magnetic anisotropy effects. The validity of this protocol has been shown by cross-checking the values obtained with values corrected by the anisotropy tensor (pers. comm. J. Morales). A large standard deviation of six specimens belonging to one sample may indicate a large anisotropy and should be rejected, assuming that effects of alteration, multidomain grains and differences in cooling rate (CR) are detected by additional acceptance criteria. Rock magnetic measurements were carried out on powdered bulk sample of about 300 mg. For the demagnetization each specimen was encapsulated in a cylindrical non-magnetic salt pellet.

### Rock magnetic and demagnetization methods

Comprehensive rock magnetic results from KRS were published in Donadini *et al*.^37^. Nevertheless, we measured three additional samples. Additionally, six DMB samples - of each sampled furnace one - and 23 samples from SIO were subjected to rock magnetic measurements (Suppl. Table S1). The analyses include thermomagnetic curves, hysteresis loops, stepwise acquisition of Isothermal Remanent Magnetization (IRM), and backfield curves. These rock magnetic measurements were performed in a Magnetic Measurements Variable Field translation Balance or Curie Balance (MMVFTB) at the National Archaeomagnetic Service (SAN) of the UNAM, Campus Morelia, Mexico. Thermomagnetic curves (magnetization vs. temperature) were measured with an applied field of 17 mT or 19 mT, and were heated to 600°C in air. Curie temperatures (*T*_*c*_) were determined using the 2nd derivative method with the program RockMagAnalyzer 1.0^60^. Backfield curves were measured while applying a saturating field up to −350 mT. IRM curves were measured up to 750 mT, and hysteresis loops up to ±700 mT. The parameters coercivity (*B*_*c*_), saturation magnetization (*M*_*s*_), and saturation remanence (*M*_*rs*_) were determined from hysteresis measurements on the raw data with RockMagAnalyzer 1.0 software. The coercivity of remanence (*B*_*cr*_) was determined from the crossing with the x-axis of the backfield curve. Finally, k-T curves were measured on 22 samples from SIO in an Agico KLY-2 kappabridge with a CS-2 heating element at the LNM laboratory at ETH Zurich. Magnetic susceptibility (k) was measured while heating the samples to 700°C in air and cooling back to room temperature.

We demagnetized 19 DMB and three SIO20 specimens with alternating fields (AF) using at least three specimens per furnace; and we applied 14 demagnetization steps from 5–100 mT. Specimens were first demagnetized up to 70 mT in a LDA5 AF demagnetizer, and the remaining remanent magnetization was measured after each step on an Agico JR6 magnetometer. The last steps up to 100 mT were demagnetized with a Schonstedt GSD-1 AF demagnetizer at the SAN Morelia. Directions of KRS specimens from each furnace were measured and analysed by Donadini *et al*.^37^.

### Archaeointensity experiments

Specimens for the archaeointensity experiments were chosen based on rock magnetic, and demagnetization measurements, as well as on other previous measurements^37,43^. We applied two different methods to obtain archaeointensities: the Thellier-Coe protocol^2,36^ and the Pseudo-Thellier protocol^11,12^.

The Thellier-Coe (TH-C) experiment was applied to 54 specimens (six from KRS, 18 from DMB, and 30 from SIO). The protocol comprises heatings to 15 increasing temperature steps, each including first a ‘zero-field’ step and then an ‘in-field’ step with an applied field *B*_*lab*_ = 35 *μ*T. In order to monitor the chemical alteration behaviour with temperature of the specimens and the presence of multi-domain grains, we applied pTRM-^2,61^ and tail-checks^62,63^ after each second temperature step. Specimens were heated in an ASC Scientific TD48 thermal demagnetizer and measured after each heating step on an Agico JR6 magnetometer. The whole experiment was performed in a shielded area. The raw data were analyzed using the palaeomagnetic program PmagPy^64^. Differences in CR between the past and laboratory cooling were taken into account with CR experiments at 480 °C (32 specimens) and 550 °C (22 specimens). The two temperatures were chosen based on the temperature step at which about 75% of their natural remanent magnetization (NRM) was lost. We followed the protocol of Chauvin *et al*.^65^ with a fast laboratory cooling of 30–45 minutes in *B*_*lab*_, obtaining a thermoremanent magnetization (TRM) *TRM*_1_, a slow cooling of 7–8 hours in *B*_*lab*_ obtaining *TRM*_2_, and again, a fast cooling in *B*_*lab*_ obtaining *TRM*_3_. We accepted the CR correction, *TRM*_1_/*TRM*_2_ if ∥*r*_1_∥≥∥*r*_2_∥, where *r*_1_ = (*TRM*_2_ − *TRM*_1_)/*TRM*_1_ and *r*_2_ = (*TRM*_3_ − *TRM*_1_)/*TRM*_1_.

The calibrated pseudo-Thellier (p-TH) experiment was first applied on a set of nine test specimens in the LNM laboratory (Zurich). First, the specimens were AF demagnetized in 17 steps - from 2.5 to 170 mT. Second, an anhysteretic remanent magnetization (ARM) was acquired at the same steps applying additionally a constant field of 35 *μ*T. Finally, the specimens were again AF demagnetized in the same 17 steps. In order to assure that the same grains that recorded the TRM also recorded the ARM, we accepted only specimens that fulfil the empirical grain size criterion 23 mT < *B*_1/2_ < 63 mT^12^. The acquired ARM was then compared to the demagnetized NRM in an Arai-type figure to obtain the slope that is proportional to the ancient geomagnetic field. To calibrate the relative intensities we used the calibration relation from de Groot *et al*.^13^. After the test specimens were successful, we performed the p-TH experiment on 22 specimens in the palaeomagnetism laboratory Fort Hoofddijk (Utrecht). In this laboratory we were able to apply AF fields up to 300 mT in the same procedure.

## Setting Up of The Reference Curves

### Data selection and relocation

The archaeointensities obtained in the present study from the TH-C and P-TH experiments were complemented with data from 0–2000 AD from within a radius of 2100 km around a central relocation point (21.0°N, 0.0°W; Fig. 7b). Furnace averages from the P-TH experiment were only accepted if they stem from more than one specimen. The additional data were (1) data from Korsimoro^43^ that were reinterpreted with the selection criteria from this study (Table 1, Table S4), (2) archaeointensities of the global database Geomagia50.v3^27^ from the North-West of Africa, (3) archaeomagnetic data that are currently not in the Geomagia database from Casas *et al*.^45^ and (4) volcanic data from the Canary islands from Sherwood^66^, Valet *et al*.^67^, de Groot *et al*.^68^, Kissel *et al*.^46^, Monster *et al*.^69^ and Calvo-Rathert *et al*.^70^ (Table S6). The complete set of data that was used to set up the reference curve contains 80 archaeointensity values. The data density is about the same during the whole 2000 years. No specific selection criteria were applied, on the one hand, because of the relatively low amount of data, and, on the other hand, because the modelling approaches take the reliability of the data into account. In particular, seven data have age uncertainties *σ*_*Age*_ > 100 yrs, and eight have standard errors *σ*_*Ba*_ > 10*μ*T. We will discuss here shortly the data points with large uncertainties. Fouzai *et al*.^71^ obtained high intensity values and respective uncertainties at 1150 AD and 436.5 AD from Tunisian kilns and slags. Although, the Arai diagrams are linear, the uncertainties are rather large due to a viscous component. To obtain the site average they fit Gaussian and Lorentzian function to the intensity distributions of the specimen estimates, leading to relatively large uncertainties. Their age has been estimated by archaeological constraints and by archaeomagnetic dating using the directional results of these samples. Casas *et al*.^45^ as well obtained one data point with relatively large uncertainty at 450 AD. This average of 15 successful specimen determinations stem from a Tunisian pottery waster. At 720 AD KRS33 exhibits a high intensity and standard error. The three specimens have been reanalysed from Kapper *et al*.^43^ and provide an average (CR corrected - not relocated) value of (65.4 ± 9.3) *μ*T. This results does not differ much from the previous analyses of (67.2 ± 6.6) *μ*T applying the soft criteria and (65.2 ± 9.4) *μ*T applying the strict criteria. Arai diagrams of the three specimens are either completely linear (KRS33-01-C, with the highest intensity of the three, Table S4) or linear until 470 °C. We therefore consider this furnace average as reliable. The next data point with rather large uncertainty was reported by Gomez-Paccard *et al*.^44^ for a Tunisian kiln dated to 1028 AD. However, this value agrees very well with another from the Canary Islands, reported by Kissel *et al*.^46^. At 1058 AD de Groot *et al*.^68^ present a data point from lava from the Canary Islands with very high intensity and very low uncertainty. They hypothesize that this site point might be wrongly dated, to about 1500 years younger. However, we keep the estimated age as it was published until a new age has been confirmed. At 1100 AD we report two data points from KRS35, one obtained with the Thellier-Coe and the other with the pseudo-Thellier method. They average to (27.6 ± 7.5) *μ*T with a *σ*_*Ba*%_ = 27.2%, which is accepted. At 1550 AD Kissel *et al*.^46^ report a very large intensity with large uncertainty for the Canary Islands. Due to the large uncertainty they do not consider this data point reliable. However, our reliable result from DMB3 supports a higher intensity around this period and lies within the uncertainty range of the data point from the Canary Islands. Finally, two additional data points, from Monster *et al*.^69^ at 1585 AD and from KRS30 at 1650 AD, have large uncertainties, but agree well with other results close in time.

All archaeointensities were relocated to the reference point to be able to compare data from a large area. The relocation procedures follow the methodology proposed by Casas and Incoronato^24^, assuming a geomagnetic field dominated by a geocentric axial dipole. The relocated intensities *Ba*_*R*_ were obtained via the virtual axial dipole moment (VADM; Barton *et al*.^72^).

1 $$B{a}_{R}=B{a}_{S}\sqrt{(1+3{\cos }^{2}{\theta }_{R})/(1+3{\cos }^{2}{\theta }_{S})}$$

where *θ*_*S*_ is the geographical colatitude of each of the archaeointensities *Ba*_*S*_, and *θ*_*R*_ is the colatitude of the relocation point.

Due to the large radius of 2100 km and the non-dipole harmonic contribution^72,73^, we thoroughly investigated if the relocation affects the data used in the present study. Casas and Incoronato^24^ studied the relocation error that arises due to the shape of the Earth and the distance from the relocation point. They calculated maximum relocation errors of 300 nT per 100 km at 2000 AD for West Africa from relocations using the IGRF-9 model. Therefore, we expect a maximum relocation error of 7.5*μ*T in 2100 km distance from the central relocation point.

For the present study we calculated the relocation error for a grid covering the circle of 2100 km from the IGRF-11^74^ and SHA.DIF.14k^26^ models for several time slots (Fig. 7b, S4). We took into account Eq. 1 and calculated the difference between each relocated value and the intensity of the central relocation point. Maximum distances of our data to the relocation point provide relocation errors between ±1.8 *μ*T on average over the epochs we tested. To take into account the relocation error, Casas and Incoronato^24^ suggested weighting data according to the distance to the relocation site. We incorporated the relocation error that was calculated from the IGRF11 model for 2000 AD as a factor in the generation of the reference curves by adding it to the error of the intensity *σ*_*Ba*_

2 $${\sigma }_{tot}=\sqrt{{\sigma }_{Ba}^{2}+{\sigma }_{rel}^{2}}$$

where *σ*_*rel*_ is the relocation error and *σ*_*tot*_ the total error.

### Establishing the reference curves

In order to obtain a good representation for the variation of the archaeointensity for the last two millennia in the North-West of Africa, we tested two methods to obtain a representative PSV curve: a smoothing spline fit and a stochastic modelling approach^35^.

For the smoothing spline fit (SSF) we applied the bootstrap method on continuous temporal penalized cubic B-spline interpolators^32^ as it was previously applied in the the study of Goguitchaishvili *et al*.^20^, i.e. using a L2-norm for the measure of misfits instead of the Huber norm used in Thebault *et al*.^32^. The bootstrap method allows generating an ensemble of PSV curves, considering a random combination of two statistic distributions for the archaeointensities and ages: (1) a Gaussian distribution with a mean and standard deviation given by the archaeointensity value and errors, respectively, (2) a uniform distribution for the ages where the minimum and maximum values are delimited by the age uncertainty. The ensemble of data *f* was fitted by a continuous temporal cubic B-splines function penalized by the second time-derivative matrix *D* to obtain the PSV curve $\widehat{f}$:

3 $$\widehat{f}={({A}^{T}WA+\lambda D)}^{-1}{A}^{T}{W}^{T}f$$

The fit function gives more weight to the intensities with low uncertainty due the diagonal matrix W, with *w*_*ii*_ = 1/*σ*^2^, where ${\sigma }^{2}={({\sigma }_{tot}/max({\sigma }_{tot}))}^{2}+{({\sigma }_{Age}/max({\sigma }_{Age}))}^{2}$ with *σ*_*Age*_ the age uncertainty. ‘*A*’ is the parameter matrix based on a set of B-splines with knots every 23.7 yrs from 0 to 2000 AD. *λ* is the damping parameter, which controls the trade-off between the fit to the data and the roughness of the intensity curve. The optimum damping parameter was obtained by searching over a range of parameters from 10^−7^ to 10^9^ and creating corresponding synthetic curves. By the comparison of the original data with the synthetic curves the root mean square (RMS) misfits were estimated. The optimum damping parameter was selected to be 100 so that the final curve is not too smooth nor too rough.

The stochastic modelling makes use of a Bayesian approach that includes a priori knowledge about the time evolution of the geomagnetic field^35^. More specifically, the method is self-consistent as it avoids the use of arbitrary basis function, such as splines, and relies instead on Gaussian process regression incorporating a priori information consistent with the statistics of the GMF derived from satellite, ground based observatories and palaeomagnetic measurements. The Huber norm^75^ is applied to deal efficiently with outliers. To account for dating uncertainties, a bootstrap method associated with a Markov Chain Monte Carlo method select models obtained from a random distribution of dates with highest probabilities. Contrary to the SSF method, the bootstrap is only performed on the data ages not on the measurement uncertainties as the latter are handled in the inversion process. From each mean model and its associated a posteriori covariance matrix obtained from the bootstrap, an ensemble of possible realizations is calculated. The final stochastic modelling curve (SMC) is presented as a pdf of intensity.

### Periodicity analysis

The multitaper power spectra were calculated using the power spectral density code of Robert Parker, based on the work of Riedel and Sidorenko^76^. We used two prolate tapers and applied them to the de-trended data. Similar results were obtained with 3 tapers, while with increasing the number of tapers, the spectrum becomes much smoother.

The wavelet analysis^77^ provides information of the time-frequency distribution, i.e., how the spectral features evolve over time. The time-frequency analysis was based on the Morlet wavelets. The number of scales is 56, and these are converted to frequencies later. With this method we confirmed the periods identified with the multitaper spectral estimates.

## Supplementary information

Suppl. Mat.

## Data Availability

All archaeointensity data will be available free for use in the Geomagia50 database (http://geomagia.gfz-potsdam.de/).
