# Author Correction: Novel insights on the geomagnetic field in West Africa from a new intensity reference curve (0–2000 AD)

**DOI:** 10.1038/s41598-020-67557-7

**Published:** 2020-06-30

**Authors:** Lisa Kapper, Vincent Serneels, Sanja Panovska, Rafael García Ruíz, Gabrielle Hellio, Lennart de Groot, Avto Goguitchaichvili, Juan Morales, Rubén Cejudo Ruíz

**Affiliations:** 1 National Archeomagnetic Service, Institute of Geophysics, Campus Morelia, Universidad Nacional Autónoma de México (UNAM), 58190 Morelia, Mexico; 2 Department of Geosciences, University of Fribourg, 1700 Fribourg, Switzerland; 3 Deutsches GeoForschungsZentrum GFZ, Helmholtz Zentrum Potsdam, 14473 TelegrafenbergPotsdam, Germany; 4 Laboratory of Planetology and Geodynamics, Nantes University, 44322 Nantes, France; 5 Paleomagnetic Laboratory Fort Hoofddijk, Utrecht University, Utrecht, 3584 CD The Netherlands

Correction to: *Scientific Reports* 10.1038/s41598-020-57611-9, published online 24 January 2020

The Acknowledgements section in this Article is incomplete.

“We thank Dr. F. Donadini for his useful comments on the draft. Sampling took place during the archaeological excavation campaigns (2012, 2013 and 2015) organized in the frame of the research project "Origine et Développement de la métallurgie du fer au Burkina Faso et en Côte d’Ivoires" funded by the Swiss-Liechtenstein Foundation for Archaeological Research Abroad (SLFA). The project was co-directed by Prof. V. Serneels (University of Fribourg, Switzerland), Dr. L. Koté and Dr. L. Simporé (University of Ouagadougou, Burkina Faso) and Dr. H.T. Kiénon-Kaboré (University of Abidjan, Ivory Coast). The archaeological excavations were performed with the help of the archaeolgy students of Abidjan and Ouagadougou, supported by a few Swiss volunteer archaeologists. We thank them all for suitable help on the field. Dr. F. Donadini was responsible for sampling at Korsimoro. V. Serneels did the sampling at Siola and Doumbala. All samples were exported for scientific studies with the permission of the relevant responsible persons in both countries. Initial funding for the archaeomagnetic study was provided by the Swiss National Science Foundation (SNSF), project 105211_144102 "Establishing paleomagnetic reference curve for W-Africa: archaeological and geophysical inference". A. Gogichaishvili acknowledges the partial financial support given by CONACYT No. 252149.”

should read:

“We thank Dr. F. Donadini for his useful comments on the draft. Sampling took place during the archaeological excavation campaigns (2012, 2013 and 2015) organized in the frame of the research project "Origine et Développement de la métallurgie du fer au Burkina Faso et en Côte d’Ivoires" funded by the Swiss-Liechtenstein Foundation for Archaeological Research Abroad (SLFA). The project was co-directed by Prof. V. Serneels (University of Fribourg, Switzerland), Dr. L. Koté and Dr. L. Simporé (University of Ouagadougou, Burkina Faso) and Dr. H.T. Kiénon-Kaboré (University of Abidjan, Ivory Coast). The archaeological excavations were performed with the help of the archaeology students of Abidjan and Ouagadougou, supported by a few Swiss volunteer archaeologists. We thank them all for suitable help on the field. Dr. F. Donadini was responsible for sampling at Korsimoro. V. Serneels did the sampling at Siola and Doumbala. All samples were exported for scientific studies with the permission of the relevant responsible persons in both countries. Initial funding for the archaeomagnetic study was provided by the Swiss National Science Foundation (SNSF), project 105211_144102 "Establishing paleomagnetic reference curve for W-Africa: archaeological and geophysical inference". A. Gogichaishvili acknowledges the partial financial support given by CONACYT No. 252149. Finally, we are grateful for the grant n° 10AC-4_194042 / 1 provided by the Swiss National Science Foundation (SNSF) to cover the article-processing charge.”

