# Supplementary material for: Novel insights on the geomagnetic field in West Africa from a new intensity reference curve (0-2000 AD)
Source: Sci Rep. 2020 Jan 24;10:1121. doi: 10.1038/s41598-020-57611-9 (PMC6981143; doi:10.1038/s41598-020-57611-9)
Supplement: Supplementary file 1 — Suppl. Mat. [file 41598_2020_57611_MOESM1_ESM.pdf]

Novel insights on the geomagnetic field in West Africa from a new intensity reference curve (0-2000 AD)

Lisa Kapper, Vincent Serneels, Sanja Panovska, Rafael Garcia Ruiz, Gabrielle Hellio, Lennart de Groot, Avto Gogichaishvili, Juan Morales, and Ruben Cejudo Ruiz

Supplementary Material

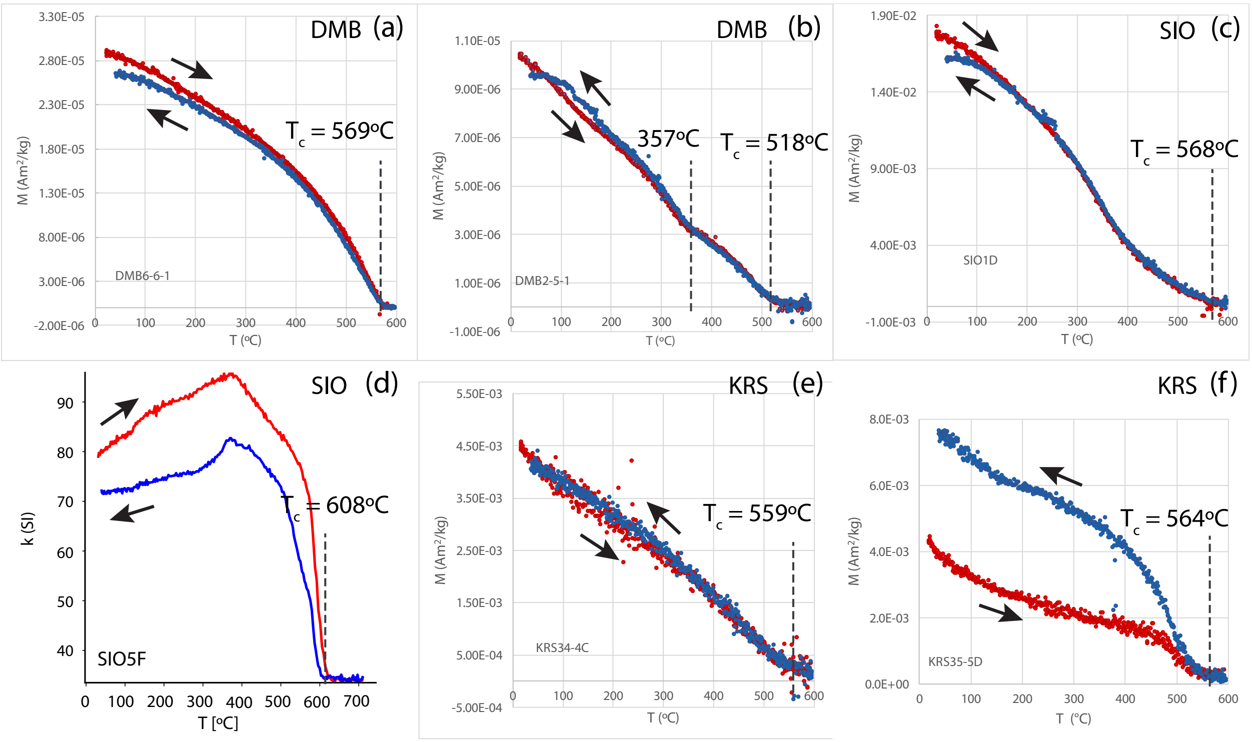

Fig. S1: Examples of typical thermomagnetic curves of samples from (a-b) Doumbala (DMB), with one and with two phases, respectively; (c-d) Siola (SIO); and (e-f) from Korsimoro (KRS). (a-c) and (e-f) are magnetization versus temperature, whereas (d) is magnetic susceptibility versus temperature.

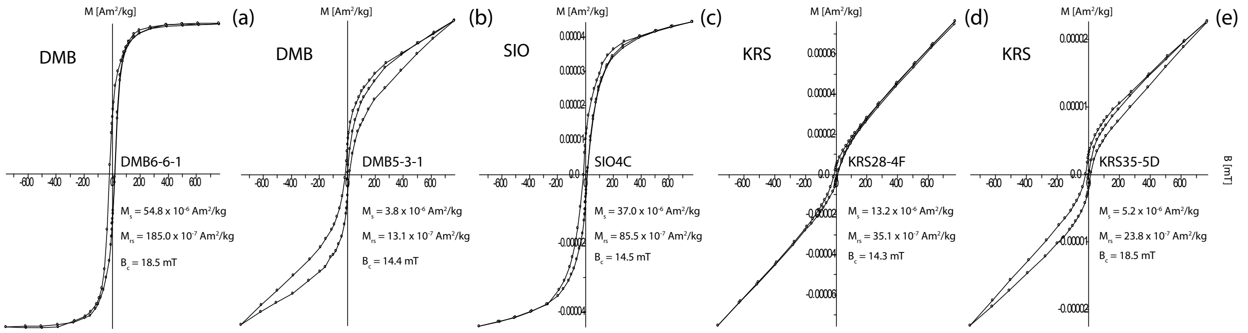

Fig. S2: Examples of typical hysteresis curves from Doumbala (DMB) with (a) narrow and (b) wasp-waisted shapes; from (c) Siola (SIO); from Korsimoro (KRS) with (d) narrow and (e) wasp-waisted shapes. The dia- and paramagnetic components were not removed.

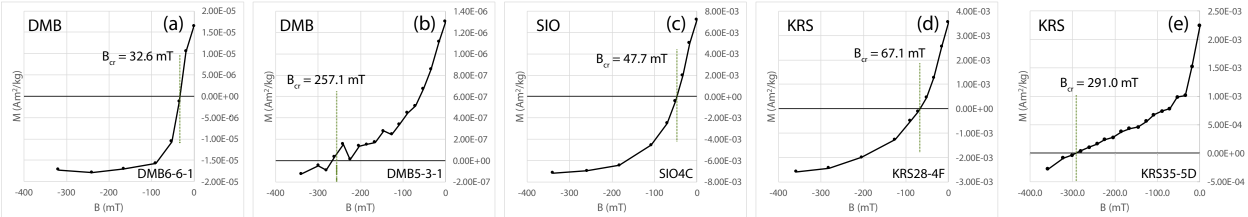

Fig. S3: Examples of backfield curves corresponding to the above shown hysteresis curves of (a-b) Doumbala (DMB), (c) Siola (SIO) and (d-e) Korsimoro (KRS).

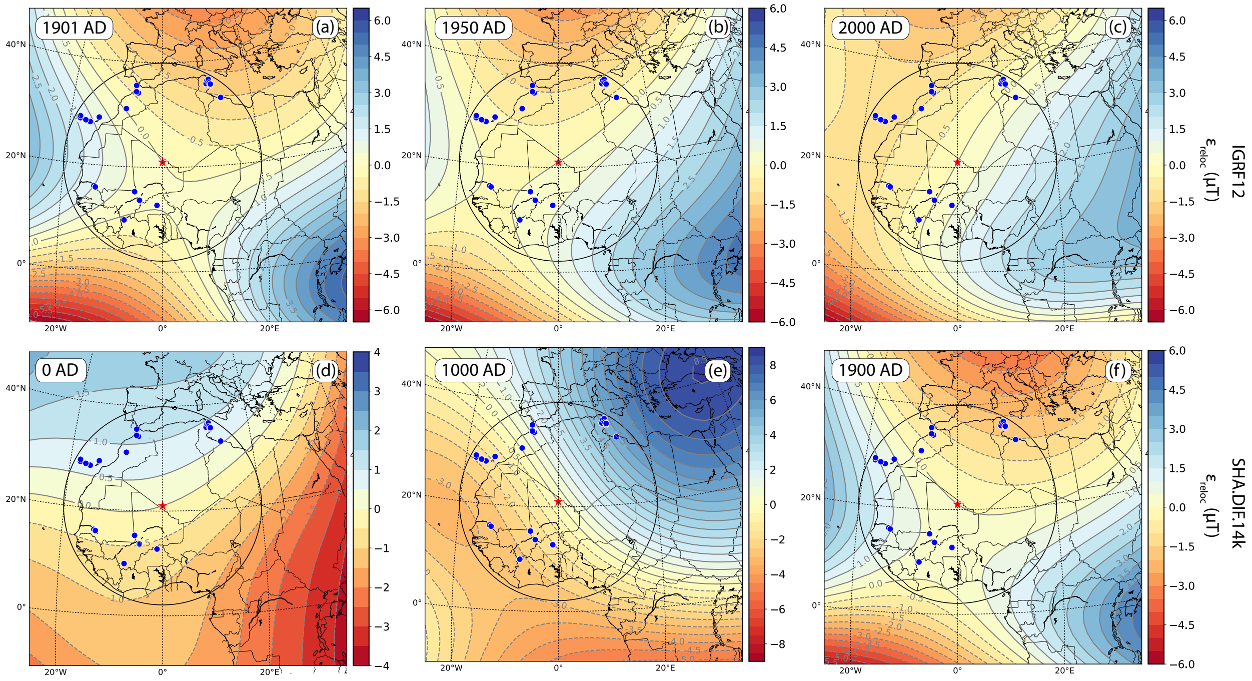

Fig. S4: Maps of the relocation error calculated using the IGRF12[1] (a-c) and the SHA.DIF.14k[2] (d-f) for different time slots. Also shown is the central relocation point (star), the locations of the data used for the reference curves (dots) and the circular area with a radius of about 2100 km.

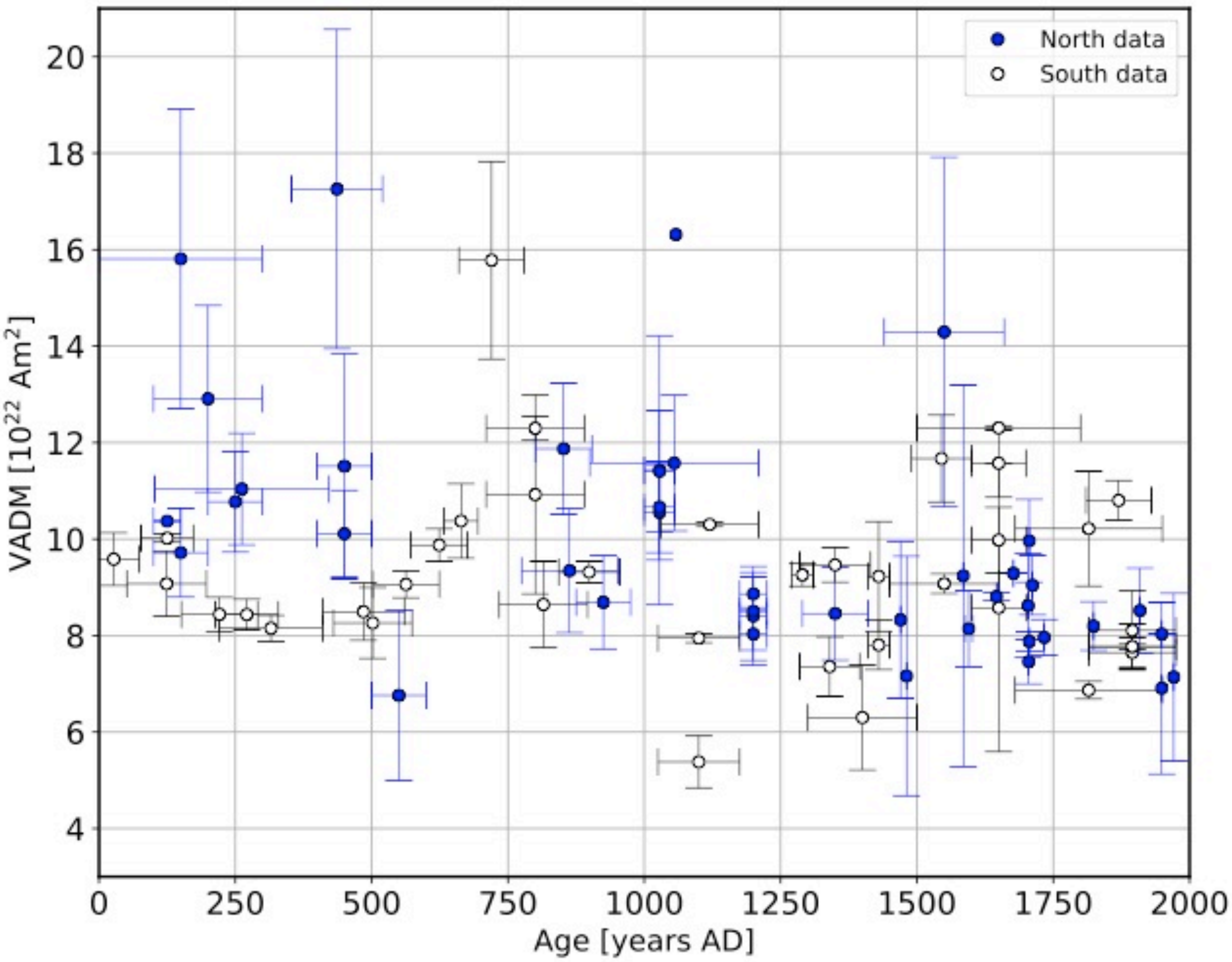

Fig. S5: Virtual Axial Dipole Moments (VADMs) of the data used for the West African reference curves. Blue (filled) dots indicate data from the Northern part of the circular area around the relocation point (Canary Islands, Morocco, Tunisia, Libya) and white (empty) dots indicate data from the Southern part (Senegal, Mali, Burkina Faso, Ivory Coast).

References:

1. Thébault, E. *et al.* International Geomagnetic Reference Field: the 12th generation. *Earth, Planets Sp.* **67,** 19 (2015).
2. Pavón-Carrasco, F. J., Osete, M. L., Torta, J. M. & De Santis, A. A geomagnetic field model for the Holocene based on archaeomagnetic and lava flow data. *Earth Planet. Sci. Lett.* **388,** 98–109 (2014).

Sample	T_c (°C)	Rev.	M_s (10^{-6} Am^2/kg)	M_{rs} (10^{-7} Am^2/kg)	B_c (mT)	B_{cr} (mT)	M_{rs}/M_s	B_{cr}/B_c
DMB1-4-1*	507/309	yes	1.9	5.0	12.7	55.3	0.27	4.36
DMB2-5-1*	518/357	yes	17.7	69.8	25.8	67.6	0.39	2.62
DMB3-4-1*	520	nearly	2.6	6.1	7.0	27.6	0.24	3.92
DMB4-3-1*	573	nearly	8.3	19.0	7.3	20.4	0.22	1.20
DMB5-3-1*	524/133	yes	3.8	13.1	14.4	257.1	0.34	17.87
DMB6-6-1*	569	nearly	54.8	185.0	18.5	32.6	0.34	1.77
**Average**	**535**		**14.8**	**49.7**	**14.3**	**76.8**	**0.30**	**5.29**
**Stdev.**	**28**		**20.4**	**70.6**	**7.1**	**90.1**	**0.07**	**6.28**
SIO1B	560/638	yes						
SIO1C	593	nearly						
SIO1D*	568	yes	34.2	93.7	12.6	35.8	0.27	2.86
SIO1D	590	no						
SIO1E	595	no						
SIO2B	635/218	nearly						
SIO2E	563/633	no						
SIO2G	587	nearly						
SIO3A	583	no						
SIO3C	632	nearly						
SIO3D	560	no						
SIO4B	597	yes						
SIO4C*	572	no	37.0	85.5	14.5	47.7	0.23	3.29
SIO4C	595	no						
SIO4E	584	yes						
SIO5D*	564	no	101.0	220.0	11.4	28.4	0.22	2.50
SIO5E	561	yes						
SIO5F	608	no						
SIO5G	526	yes						
SIO6G	596	no						
SIO6H	605	no						
SIO6I	626	no						
SIO7B	558	no						
SIO7D	562	no						
SIO7G	588	no						
SIO20*	559	yes	0.7	1.2	9.2	21.5	0.18	2.33
**Average**	**583**		**43.2**	**100.1**	**11.9**	**33.4**	**0.23**	**2.75**
**Stdev.**	**26**		**41.9**	**90.2**	**2.2**	**11.2**	**0.04**	**0.43**
KRS28-4F*	559	no	13.2	35.1	14.3	67.1	0.27	4.69
KRS34-4C*	584	yes	7.4	18.2	9.4	63.0	0.25	6.72
KRS35-5D*	564	no	5.2	23.8	18.5	291.0	0.46	15.75
**Average**	**569**		**8.6**	**25.7**	**14.1**	**140.3**	**0.33**	**9.05**
**Stdev.**	**13**		**4.1**	**8.6**	**4.6**	**130.5**	**0.12**	**5.89**

Tab. S1: Rock magnetic properties of Doumbala (DMB), Siola (SIO) and Korsimoro (KRS) samples. Shown are the Curie temperature T_c, Rev. indicates the reversibility of the heating and cooling curve in the thermomagnetic experiment; M_s the saturation magnetization; M_{rs} the remanence; B_c the coercivity and B_{cr} the coercivity of remanence. Samples with a star were measured on the Curie Balance, while the others were measured on the kappabridge.

Tab. S2: Results of the Thellier-Coe intensity experiment of Doumbala (DMB), Korsimoro (KRS) and Siola (SIO) specimen. $Step_{min}$ and $Step_{max}$ are the beginning and endpoint of the best fit slope, N is the number of points used for the best fit. Ba is the archaeointensity obtained from the best fit and σ_{Ba} is the standard error of the slope. Furthermore, β is the ratio of the standard error of the slope to the absolute value of the slope; FRAC the fraction of remanence; GAP-MAX the maximum gap statistic between two consecutive data points; DANG the deviation of the angle, as to ensure that the characteristic component was chosen; and MAD the maximum angular deviation. Rejected specimens are marked with a star.

	Specimen	$Step_{min}$	$Step_{max}$	N	Ba (μT)	σ_{Ba} (μT)	β	FRAC	GAP-MAX	DANG (°)	MAD (°)
1	DMB1-12-07	280	480	7	33.80	0.03	0.03	0.64	0.29	4.96	3.02
2	DMB1-12-08	150	400	8	32.30	0.03	0.03	0.69	0.26	2.45	4.15
3	DMB1-12-09	250	370	5	32.50	0.05	0.05	0.62	0.39	1.62	1.63
4	DMB1-12-10	100	480	11	31.40	0.03	0.03	0.69	0.19	0.77	4.06
5	DMB1-12-11*	100	370	8	39.90	0.35	0.30*	0.62	0.31	2.04	4.55
6	DMB1-12-12	100	520	12	32.60	0.02	0.02	0.80	0.20	0.73	2.89
7	DMB2-52-13	0	480	12	32.10	0.02	0.02	0.96	0.21	0.79	2.88
8	DMB2-52-14	100	480	11	32.40	0.03	0.03	0.89	0.37	0.30	3.36
9	DMB2-52-15	100	440	10	31.90	0.03	0.04	0.73	0.50	1.95	4.99
10	DMB2-52-16	310	440	5	32.20	0.04	0.05	0.67	0.30	2.40	4.08
11	DMB2-52-17	0	550	14	32.10	0.04	0.04	0.99	0.23	1.84	3.32
12	DMB2-52-18	150	480	10	32.10	0.03	0.03	0.87	0.34	0.73	4.86
13	DMB3-24-19	0	400	10	38.60	0.02	0.01	0.82	0.29	1.98	3.53
14	DMB3-24-20	310	480	6	38.50	0.04	0.04	0.75	0.39	1.02	1.51
15	DMB3-24-21	150	370	7	37.40	0.04	0.03	0.85	0.35	0.94	3.17
16	DMB3-24-22	0	480	12	38.80	0.02	0.02	0.99	0.28	0.70	1.75
17	DMB3-24-23	150	480	10	38.70	0.03	0.02	0.97	0.28	0.93	2.00
18	DMB3-24-24	0	340	8	39.80	0.04	0.04	0.65	0.44	2.15	3.44
	**Average**	**126**	**451**	**9**	**34.54**	**0.03**	**0.03**	**0.80**	**0.31**	**1.54**	**3.21**
	**Stdev.**	**109**	**57**	**3**	**3.18**	**0.01**	**0.01**	**0.07**	**0.09**	**1.11**	**1.05**
1	KRS28-01-01	100	520	12	49.30	0.10	0.07	0.63	0.17	1.70	3.80
2	KRS28-01-02	150	480	10	48.70	0.07	0.05	0.64	0.21	3.05	3.98

	Specimen	$Step_{min}$	$Step_{max}$	N	Ba (μT)	σ_{Ba} (μT)	β	FRAC	GAP-MAX	DANG (°)	MAD (°)
3	KRS28-01-03	150	550	12	43.40	0.05	0.04	0.64	0.15	1.74	3.38
4	KRS28-01-04	150	550	12	47.40	0.06	0.05	0.63	0.16	1.04	3.55
5	KRS28-01-05	0	580	15	52.10	0.08	0.05	0.95	0.13	1.25	4.66
6	KRS28-01-06	0	550	14	54.00	0.07	0.05	0.77	0.15	3.21	3.80
	**Average**	**92**	**538**	**13**	**49.15**	**0.07**	**0.05**	**0.71**	**0.16**	**2.00**	**3.86**
	**Stdev.**	**74**	**34**	**2**	**3.70**	**0.02**	**0.01**	**0.13**	**0.03**	**0.92**	**0.44**
1	SIO1BA-25*	150	480	10	53.60	0.15	0.10*	0.64	0.23	5.19*	6.13*
2	SIO1BA-26*	370	520	5	58.90	0.1	0.06	0.42*	0.58	1.91	2.91
3	SIO1BA-27	340	580	8	45.90	0.07	0.05	0.61	0.27	1.04	2.59
4	SIO1BA-28	150	550	12	51.40	0.07	0.05	0.88	0.26	0.61	4.62
5	SIO1BA-29	250	520	9	38.50	0.07	0.06	0.63	0.22	3.29	4.87
6	SIO1BA-30*	250	550	10	47.90	0.11	0.08	0.58*	0.16	4.19	6.38*
7	SIO3CA-31*	150	440	9	39.20	0.11	0.10*	0.76	0.19	3.32	13.80*
8	SIO3CA-32*	150	440	9	37.20	0.12	0.11*	0.76	0.18	11.99*	18.30*
9	SIO3CA-33*	280	480	7	41.80	0.31	0.26*	0.61	0.31	2.65	13.38*
10	SIO3CA-34*	280	480	7	40.10	0.32	0.28*	0.54*	0.29	3.09	14.45*
11	SIO3CA-35*	250	440	7	36.70	0.16	0.16*	0.66	0.24	8.62*	16.97*
12	SIO3CA-36*	150	340	6	39.30	0.07	0.06	0.44*	0.39	11.35*	13.50*
13	SIO4BA-37	370	580	7	43.80	0.06	0.05	0.68	0.31	1.92	2.41
14	SIO4BA-38	400	580	6	48.10	0.09	0.06	0.64	0.28	2.00	4.10
15	SIO4BA-39	100	620	15	51.40	0.05	0.03	0.97	0.27	1.46	3.98
16	SIO4BA-40	310	580	9	48.30	0.06	0.04	0.72	0.25	2.51	4.95
17	SIO4BA-41	310	580	9	48.30	0.05	0.04	0.71	0.28	1.91	2.71
18	SIO4BA-42	200	550	11	55.00	0.04	0.02	0.68	0.25	1.35	4.37
19	SIO5GA-43*	100	480	11	52.00	0.09	0.06	0.59*	0.25	2.79	10.2
20	SIO5GA-44	340	620	9	43.50	0.05	0.04	0.80	0.23	3.88	4.68
21	SIO5GA-45	250	520	9	48.10	0.05	0.03	0.74	0.29	2.73	3.28
22	SIO5GA-46	370	620	8	45.40	0.08	0.06	0.64	0.27	1.99	4.01
23	SIO5GA-47	370	520	5	46.60	0.06	0.04	0.61	0.34	1.87	2.85
24	SIO5GA-48	310	520	7	44.80	0.07	0.05	0.64	0.29	3.78	4.99

	Specimen	$Step_{min}$	$Step_{max}$	N	Ba (μT)	σ_{Ba} (μT)	β	FRAC	GAP-MAX	DANG (°)	MAD (°)
25	SIO5GC-49*	280	520	8	37.20	0.09	0.08	0.45*	0.32	5.00	9.39*
26	SIO5GC-50	310	550	8	47.00	0.06	0.05	0.64	0.25	1.34	3.31
27	SIO5GC-51	310	550	8	43.10	0.08	0.06	0.61	0.31	0.10	3.90
28	SIO5GC-52	400	550	5	40.40	0.08	0.07	0.76	0.55	0.36	0.40
29	SIO5GC-53*	340	520	6	38.10	0.12	0.11*	0.51*	0.5	5.79*	4.84
30	SIO5GC-54*	340	550	7	33.20	0.04	0.04	0.21*	0.32	7.10*	15.75*
	**Average**	**299**	**564**	**9**	**46.54**	**0.06**	**0.05**	**0.70**	**0.29**	**1.89**	**3.65**
	**Stdev.**	**85**	**35**	**2**	**4.07**	**0.01**	**0.01**	**0.10**	**0.07**	**1.10**	**1.20**

Tab. S3: Results of the pseudoThellier intensity experiment of Doumbala (DMB), Korsimoro (KRS) and Siola (SIO) specimen. $B_{1/2ARM}$ is the alternating field strength that imparts half of the saturated anhysteretic remanent magnetization (ARM); Ba is the archaeointensity obtained from the linear fit to the pseudoThellier (pTH) slope and by calibration; the pTh slope wass obtained from an Arai plot of the natural remament magnetization (NRM) remaining against the ARM acquired; R^2 indicates the goodness of the fit; and $Step_{min}$ and $Step_{max}$ define the beginning and endpoint pTh slope of the linear fit slope. Rejected specimens are marked with a star. The specimen marked with '1' was rejected because its Arai diagram is curved. Specimens marked with '2' were measured in Zurich.

	Specimen	$B_{1/2ARM}$ (mT)	Ba (μT)	pTh slope	R^2	$Step_{min}$ (mT)	$Step_{max}$ (mT)
1	DMB1-1-3	30.7	27.2	1.63	0.999	20	70
2	DMB1-1-4*	14.2*					
3	DMB1-1-5	37.9	35.2	2.67	0.995	20	70
4	DMB1-1-6	36.1	34.3	2.55	0.995	20	70
5	DMB1-1-7	26.0	34.2	2.53	0.997	20	70
6	DMB2-5-3	28.1	29.5	1.93	0.999	15	70
7	DMB2-5-4	29.2	29.6	1.94	0.999	15	70
8	DMB2-5-5	42.4	32.9	2.37	0.996	15	70
9	DMB2-5-6*	17.7*					
10	DMB2-5-7	23.8	33.5	2.45	1.000	15	70
11	DMB3-4-2*	13.1*					
12	DMB3-4-3*	9.1*					
13	DMB3-4-5*	14.4*					
14	DMB3-4-6*	14.3*					
15	DMB3-4-8*	19.8*					
16	DMB4-3-2*	14.1*					
17	DMB4-3-3*	14.2*					
18	DMB4-3-6*	15.3*					
19	DMB4-3-8*	13.8*					
20	DMB6-6-2*	21.6*					
21	DMB6-6-3	30.4	38.6	3.11	0.997	15	60
22	DMB6-6-5*	15.4*					

	Specimen	$B_{1/2ARM}$ (mT)	Ba (μT)	pTh slope	R^2	$Step_{min}$ (mT)	$Step_{max}$ (mT)
23	DMB6-6-7	30.9	36.4	2.82	0.999	15	60
24	DMB6-6-8	27.0	39.6	3.23	0.996	15	60
	**Average**	**31.1**	**33.7**	**2.48**	**0.997**	**17**	**67**
	**Stdev.**	**5.6**	**3.8**	**0.50**	**0.002**	**3**	**5**
1	KRS05-03-O*	19.6*					
2	KRS05-04-M*	18.0*					
3	KRS05-09-I*	11.6*					
4	KRS05-09-R*	14.6*					
5	KRS06-05-D	29.6	47.8	4.31	0.996	15	80
6	KRS06-05-G*	16.9*					
7	KRS06-07-D*	15.0*					
8	KRS06-07-N*[2]	-23.8*	37.8	2.50	0.982	10	170
9	KRS06-07-T*	16.4*					
10	KRS10-02-C*	21.1*					
11	KRS10-04-H[2]	39.7	32.0	1.81	0.986	10	170
12	KRS10-06-F*	19.4*					
13	KRS13-02-F*	15.7*					
14	KRS13-04-C*	17.2*					
15	KRS13-05-F*	15.3*					
16	KRS13-05-G*	14.9*					
17	KRS21-02-C*	87.2*					
18	KRS21-02-H	46.3	22.6	1.04	0.989	15	100
19	KRS21-09-B*	87.7*					
20	KRS21-09-C	47.0	29.6	1.94	0.995	15	100
21	KRS23-04-B[2]	59.7	37.6	2.48	0.998	10	170
22	KRS23-04-C	32.0	23.9	1.20	1.000	15	80
23	KRS23-05-C	47.0	31.9	2.24	0.995	15	80
24	KRS23-06-A	27.4	38.6	3.11	1.000	15	80
25	KRS23-06-C*[2]	8.7*	44.0	3.23	0.999	10	170
26	KRS23-06-G	35.6	32.9	2.37	0.998	15	80

	Specimen	$B_{1/2ARM}$ (mT)	Ba (μT)	pTh slope	R^2	$Step_{min}$ (mT)	$Step_{max}$ (mT)
27	KRS23-07-J	34.3	30.9	2.12	0.997	15	80
28	KRS28-01-I	27.1	33.8	2.49	0.999	15	100
29	KRS28-01-H*	12.6*					
30	KRS28-01-N*	12.3*					
31	KRS28-03-M*	11.9*					
32	KRS28-04-L*	18.1*					
33	KRS30-04-B*	12.5*					
34	KRS30-04-C*	13.0*					
35	KRS30-08-B	37.3	28.4	1.78	0.995	15	80
36	KRS30-08-F	30.6	27.0	1.61	0.999	15	80
37	KRS30-08-G*	18.7*					
38	KRS30-08-M[2]	27.9	51.2	4.09	0.997	10	170
39	KRS33-01-E*	16.3*					
40	KRS33-01-F*	16.3*					
41	KRS33-01-G*	20.4*					
42	KRS33-03-C*	13.9*					
43	KRS33-04-A*	14.9*					
44	KRS34-03-D*	21.2*					
45	KRS34-05-A*[2]	10.6*	25.7	1.06	0.992	10	170
46	KRS34-06-A*	21.4*					
47	KRS34-06-B*	20.7*					
48	KRS34-06-G[2]	12.1*	26.7	1.20	0.979	10	170
49	KRS34-06-M*	17.8*					
50	KRS34-06-N*	17.7*					
51	KRS35-01-B*[1]	54.0	25.8	1.45	0.950	15	80
52	KRS35-02-B*	21.8*					
53	KRS35-02-C*	14.2*					
54	KRS35-05-H	28.2	20.6	0.77	0.998	15	80
55	KRS35-08-D	62.9	24.0	1.22	0.998	15	80
	**Average**	**39.2**	**31.7**	**2.12**	**0.994**	**14**	**99**

	Specimen	$B_{1/2ARM}$ (mT)	Ba (μT)	pTh slope	R^2	$Step_{min}$ (mT)	$Step_{max}$ (mT)
	**Stdev.**	**12.0**	**8.6**	**1.02**	**0.012**	**2**	**35**
1	SIO1A-1	32.8	28.3	1.77	0.984	10	60
2	SIO1B-1	23.2	27.1	1.62	0.998	10	60
3	SIO1B-2*	21.0*					
4	SIO1B-3*	18.1*					
5	SIO1C-1*	21.2*					
6	SIO3B-1	27.9	44.2	3.84	0.999	10	100
7	SIO3B-2*	18.6*					
8	SIO3C-1*	18.6*					
9	SIO3E-1*	16.1*					
10	SIO4B-1*	19.5*					
11	SIO4B-2*	20.2*					
12	SIO4B-3*	21.7*					
13	SIO4C-1*	18.7*					
14	SIO4E-1*	21.8*					
15	SIO5A-1	39.3	38.8	3.14	0.998	15	100
16	SIO5D-1*	15.2*					
17	SIO5G-1*	13.2*					
18	SIO5G-2*	12.8*					
19	SIO5G-3*	13.8*					
20	SIO20-1-2*	15.0*					
21	SIO20-1-3*	16.3*					
22	SIO20-1-4*	15.1*					
23	SIO20-1-5*	15.8*					
24	SIO20-1-8*	15.7*					
	**Average**	**30.8**	**34.6**	**2.59**	**0.995**	**11**	**80**
	**Stdev.**	**6.9**	**8.3**	**1.07**	**0.007**	**3**	**23**

Tab. S4: Reanalysis of Korsimoro (KRS) specimens from Kapper et al[1]. $Step_{min}$ and $Step_{max}$ are the beginning and endpoint of the best fit slope, N is the number of points used for the best fit. Ba is the archaeointensity obtained from the best fit and σ_{Ba} is the standard error of the slope. Furthermore, β is the ratio of the standard error of the slope to the absolute value of the slope; FRAC the fraction of remanence; GAP-MAX the maximum gap statistic between two consecutive data points; DANG the deviation of the angle, as to ensure that the characteristic component was chosen; and MAD the maximum angular deviation. Specimens with a star were rejected.

	Specimen	$Step_{min}$	$Step_{max}$	N	Ba (μT)	σ_{Ba} (μT)	β	FRAC	GAP-MAX	DANG (°)	MAD (°)
1	KRS05-01-H*	0	260	6	52.30	0.25	0.16	0.27*	0.30	10.55*	10.36*
2	KRS05-01-Q*	0	290	7	57.20	0.48	0.30	0.36*	0.39	9.59*	12.08*
3	KRS05-03-C*	100	470	12	61.10	0.17	0.09	0.82	0.16	6.00*	4.45
4	KRS05-03-D*	260	470	8	56.50	0.27	0.17*	0.66	0.28	1.69	4.64
5	KRS05-04-B*	100	470	12	70.10	0.18	0.09	0.55*	0.41	12.25*	10.66*
6	KRS05-04-J	100	500	13	53.00	0.09	0.06	0.74	0.15	4.19	3.48
7	KRS05-04-K	200	470	10	51.60	0.04	0.03	0.62	0.18	2.74	3.09
8	KRS05-04-L*	100	350	8	55.40	0.13	0.08	0.52*	0.25	6.69*	7.75*
9	KRS05-08-F*	0	290	7	55.80	0.10	0.06	0.36*	0.27	5.59*	5.73*
10	KRS05-08-G*	100	440	11	50.30	0.16	0.11*	0.66	0.16	4.34	4.45
11	KRS05-08-K	200	500	11	53.40	0.10	0.06	0.71	0.18	1.99	1.99
12	KRS05-08-L	200	470	10	54.40	0.08	0.05	0.60	0.17	2.61	2.65
13	KRS05-09-I	100	500	13	53.70	0.09	0.06	0.78	0.17	2.43	2.48
14	KRS05-09-J*	100	440	11	49.20	0.16	0.11*	0.62	0.20	4.34	5.05*
15	KRS05-09-K*	0	410	11	59.50	0.18	0.10*	0.61	0.18	5.70*	7.21*
16	KRS05-09-M	0	500	14	55.60	0.09	0.06	0.81	0.15	1.56	2.84
17	KRS06-05-F	230	500	10	54.60	0.08	0.05	0.63	0.20	1.02	1.84
18	KRS06-07-K	100	470	12	38.70	0.06	0.05	0.66	0.15	4.1	3.42
19	KRS06-07-O	100	470	12	42.60	0.06	0.05	0.64	0.16	3.49	3.14
20	KRS07-04-F	290	540	9	16.80	0.03	0.06	0.64	0.22	3.29	3.92
21	KRS07-04-N*	200	500	11	19.80	0.06	0.10*	0.65	0.16	3.39	4.57
22	KRS07-04-T*	200	470	10	19.50	0.06	0.11*	0.50*	0.15	4.30	5.00
23	KRS10-02-A*	150	350	7	36.10	0.04	0.04	0.50*	0.22	3.62	4.74

	Specimen	$Step_{min}$	$Step_{max}$	N	Ba (μT)	σ_{Ba} (μT)	β	FRAC	GAP-MAX	DANG (°)	MAD (°)
24	KRS10-03-D	100	440	11	35.10	0.06	0.06	0.73	0.27	0.97	3.53
25	KRS10-03-G	200	440	9	34.10	0.03	0.03	0.60	0.19	1.10	1.76
26	KRS10-03-H	100	350	8	33.90	0.03	0.03	0.64	0.26	3.06	2.48
27	KRS10-04-F	150	500	12	29.00	0.02	0.03	0.65	0.14	2.39	2.45
28	KRS10-04-G	100	470	12	31.10	0.02	0.02	0.60	0.16	3.81	2.54
29	KRS10-04-I*	150	410	9	33.60	0.06	0.07	0.37*	0.24	3.49	3.58
30	KRS10-06-G*	200	440	9	36.00	0.11	0.11*	0.51*	0.42	0.83	2.91
31	KRS13-06-E	0	410	11	53.80	0.10	0.07	0.71	0.14	0.69	3.49
32	KRS13-06-F	0	380	10	53.50	0.12	0.08	0.65	0.17	3.55	4.68
33	KRS13-06-G*	150	380	8	52.90	0.16	0.10*	0.52*	0.20	1.21	7.10*
34	KRS17-04-E*	150	410	9	39.90	0.11	0.10*	0.52*	0.22	4.28	3.37
35	KRS17-04-F	0	440	12	39.30	0.08	0.07	0.68	0.12	4.96	3.72
36	KRS17-04-G*	100	380	9	39.00	0.08	0.07	0.52*	0.17	5.06*	3.85
37	KRS1A-01-B*	100	350	8	62.30	0.34	0.19*	0.41*	0.29	27.60*	4.00
38	KRS1A-02-A*	100	540	14	63.30	0.32	0.18*	0.92	0.17	12.27*	4.78
39	KRS1A-03-A*	150	540	13	61.60	0.43	0.24*	0.86	0.18	17.93*	3.55
40	KRS1B-03-A*	150	410	9	28.80	0.19	0.23*	0.43*	0.20	18.89*	4.15
41	KRS1D-04-A*	100	410	10	30.70	0.08	0.09	0.54*	0.16	12.39*	10.48*
42	KRS1E-03-A*	100	410	10	34.00	0.08	0.08	0.64	0.15	10.09*	6.37*
43	KRS1E-05-A*	100	440	11	38.40	0.13	0.12*	0.66	0.16	7.45*	4.40
44	KRS1F-01-B*	100	380	9	11.30	0.08	0.23*	0.18*	0.20	29.33*	11.99*
45	KRS1F-02-A*	100	350	8	13.00	0.06	0.17*	0.17*	0.24	14.60*	14.92*
46	KRS21-02-A*	100	500	13	38.50	0.14	0.12	0.65	0.66	1.84	1.16
47	KRS21-02-E*	100	580	15	35.00	0.15	0.15*	0.54*	0.62*	1.61	1.29
48	KRS21-02-F*	100	540	14	36.00	0.16	0.15	0.42	0.53	2.27	1.72
49	KRS21-09-A	100	580	15	33.80	0.06	0.06	0.69	0.28	1.12	1.95
50	KRS21-09-F*	0	320	8	32.20	0.11	0.12*	0.96	0.56	3.63	5.84*
51	KRS21-09-H*	100	320	7	46.30	0.41	0.31*	0.29*	0.72*	3.00	1.29
52	KRS23-04-A	150	540	13	32.60	0.01	0.01	0.73	0.34	3.86	4.12
53	KRS23-04-C	150	540	13	36.00	0.02	0.02	0.81	0.32	1.44	2.06

	Specimen	$Step_{min}$	$Step_{max}$	N	Ba (μT)	σ_{Ba} (μT)	β	FRAC	GAP-MAX	DANG (°)	MAD (°)
54	KRS23-04-H*	100	440	11	43.90	0.12	0.09	0.36*	0.21	7.76*	4.06
55	KRS23-04-I	0	350	9	37.70	0.03	0.03	0.70	0.40	2.89	2.59
56	KRS23-05-A	290	540	9	26.20	0.06	0.08	0.70	0.28	1.29	2.60
57	KRS23-05-F	0	380	10	39.80	0.04	0.04	0.69	0.22	1.85	3.04
58	KRS23-06-D	380	620	8	45.00	0.02	0.02	0.70	0.20	0.37	1.49
59	KRS23-06-I	350	580	8	44.00	0.03	0.03	0.63	0.21	1.35	1.43
60	KRS23-06-N*	150	440	10	39.50	0.12	0.10*	0.61	0.21	4.45	9.02*
61	KRS23-06-S*	150	470	11	40.40	0.11	0.09*	0.72	0.22	4.21	3.45
62	KRS23-07-G*	150	540	13	39.00	0.09	0.08	0.86	0.15	5.45*	6.05*
63	KRS23-07-I	0	350	9	46.80	0.06	0.04	0.64	0.19	1.74	4.96
64	KRS24-02-K*	100	410	10	37.60	0.05	0.05	0.61	0.18	4.25	9.44*
65	KRS24-02-L*	100	350	8	44.60	0.10	0.08	0.51*	0.21	3.51	12.24*
66	KRS24-05-F*	200	500	11	24.70	0.07	0.10	0.68	0.27	21.53*	12.02*
67	KRS24-05-G*	100	410	10	43.60	0.07	0.05	0.35*	0.22	6.58*	5.89*
68	KRS24-05-K*	100	350	8	41.40	0.07	0.06	0.34*	0.33	14.13*	4.12
69	KRS24-05-V	290	580	10	45.60	0.05	0.04	0.64	0.18	1.39	3.25
70	KRS24-05-Y	0	500	14	45.30	0.04	0.03	0.68	0.14	4.16	3.79
71	KRS24-06-I*	0	410	11	42.50	0.06	0.05	0.42*	0.26	2.26	3.05
72	KRS24-06-J*	100	380	9	42.60	0.08	0.07	0.38*	0.23	3.60	8.19*
73	KRS2A-03-A*	150	440	10	44.30	0.12	0.10*	0.51*	0.15	4.85	5.60*
74	KRS2A-04-B*	100	380	9	35.60	0.08	0.08	0.40*	0.17	5.16*	8.59*
75	KRS2A-05-A*	150	440	10	49.80	0.16	0.11*	0.59*	0.19	1.50	4.89
76	KRS2B-04-B*	150	410	9	40.50	0.10	0.09*	0.49*	0.17	4.14	6.49*
77	KRS2B-05-A*	200	350	6	24.00	0.15	0.23*	0.27*	0.32	23.75*	15.95*
78	KRS2D-01-A*	100	380	9	35.50	0.12	0.12*	0.54*	0.23	5.98*	6.66*
79	KRS2D-02-B*	150	440	10	33.80	0.13	0.14*	0.49*	0.22	3.65	8.57*
80	KRS30-08-A*	100	230	4*	45.40	0.19	0.14*	0.21*	0.40	7.58	3.94
81	KRS30-08-C	0	350	9	45.80	0.06	0.05	0.69	0.21	3.47	3.13
82	KRS30-08-D	0	290	7	41.20	0.03	0.03	0.62	0.24	2.07	1.72
83	KRS33-01-A	100	500	13	59.30	0.06	0.03	0.68	0.15	1.72	2.19

	Specimen	$Step_{min}$	$Step_{max}$	N	Ba (μT)	σ_{Ba} (μT)	β	FRAC	GAP-MAX	DANG (°)	MAD (°)
84	KRS33-01-B	0	440	12	68.20	0.04	0.02	0.61	0.15	2.40	3.13
85	KRS33-01-C	260	540	10	78.90	0.07	0.03	0.62	0.29	0.11	0.91
86	KRS34-03-B*	100	440	11	39.50	0.07	0.07	0.39*	0.17	11.91*	10.29*
87	KRS34-03-C*	200	470	10	39.40	0.06	0.06	0.56*	0.35	6.69*	4.42
88	KRS34-05-C*	150	500	12	28.90	0.08	0.10*	0.62	0.15	7.16*	15.37*
89	KRS34-05-D*	100	410	10	38.00	0.09	0.08	0.51*	0.16	3.97	9.00*
90	KRS34-05-E	200	620	14	40.70	0.04	0.03	0.78	0.23	3.91	3.39
91	KRS34-05-F	290	620	11	38.60	0.04	0.04	0.63	0.16	3.07	2.77
92	KRS34-05-N	380	620	8	40.70	0.06	0.06	0.60	0.40	2.10	0.79
93	KRS34-06-C*	150	440	10	40.70	0.07	0.06	0.66	0.16	2.06	5.75*
94	KRS34-06-D	0	500	14	40.80	0.03	0.03	0.79	0.11	3.26	4.99
95	KRS34-06-E*	150	440	10	40.60	0.10	0.09*	0.63	0.19	3.40	7.33*
96	KRS34-06-F	0	470	13	41.70	0.03	0.02	0.63	0.14	0.73	2.34
97	KRS34-06-I	0	440	12	42.30	0.02	0.02	0.67	0.12	4.04	4.88
98	KRS35-01-A	0	380	10	39.40	0.05	0.05	0.68	0.36	4.91	4.06
99	KRS35-01-C*	100	410	10	32.80	0.07	0.07	0.30*	0.23	1.87	3.89
100	KRS35-01-D	150	540	13	34.70	0.03	0.03	0.62	0.36	0.32	1.26
101	KRS35-01-E	100	540	14	34.60	0.02	0.02	0.82	0.21	1.70	2.86
102	KRS35-02-D*	0	410	11	34.50	0.05	0.05	0.52*	0.28	4.60	6.55*
103	KRS35-02-G*	100	410	10	34.80	0.07	0.07	0.40*	0.22	0.96	7.74*
104	KRS35-05-A	100	620	16	36.90	0.06	0.06	0.74	0.57	1.52	1.41
105	KRS35-05-B	100	540	14	33.20	0.04	0.04	0.76	0.22	0.88	1.65
106	KRS35-05-E*	0	350	9	30.30	0.04	0.05	0.48*	0.37	4.47	3.37
107	KRS35-05-F	0	260	6	34.90	0.04	0.04	0.71	0.44	2.32	2.75
108	KRS35-05-I	100	580	15	35.30	0.04	0.04	0.72	0.23	0.43	1.17
109	KRS35-07-A*	100	380	9	35.90	0.10	0.10*	0.34*	0.28	7.71	9.11*
110	KRS35-07-F	0	410	11	37.00	0.05	0.05	0.61	0.26	3.44	3.68
111	KRS3A-04-A*	100	410	10	36.00	0.10	0.09	0.64	0.21	6.27*	5.97
112	KRS3B-04-A*	100	410	10	35.40	0.13	0.12*	0.62	0.31	5.03*	4.72
113	KRS3B-05-B*	100	410	10	34.10	0.11	0.11*	0.65	0.22	8.22*	5.09*

	Specimen	$Step_{min}$	$Step_{max}$	N	Ba (μT)	σ_{Ba} (μT)	β	FRAC	GAP-MAX	DANG (°)	MAD (°)
114	KRS3D-03-A	0	410	11	36.20	0.07	0.07	0.75	0.21	4.78	3.54
115	KRS3E-02-B*	100	470	12	51.90	0.09	0.06	0.50*	0.19	10.07*	4.19
116	KRS3E-04-A*	100	350	8	24.30	0.04	0.06	0.33*	0.18	17.90*	7.06*
117	KRS4B-01-A	100	580	15	45.60	0.03	0.02	0.69	0.23	2.65	4.34
118	KRS4B-02-B*	100	380	9	45.20	0.10	0.08	0.35*	0.56	2.58	5.32*
119	KRS4D-04-A*	100	410	10	43.00	0.10	0.08	0.41*	0.35	4.22	11.82*
120	KRS4E-05-A*	0	580	16	34.70	0.02	0.03	0.84	0.16	1.77	5.72*
	**Average**	**115**	**483**	**11**	**42.46**	**0.05**	**0.04**	**0.68**	**0.22**	**2.38**	**2.83**
	**Stdev.**	**114**	**89**	**2**	**10.73**	**0.03**	**0.02**	**0.06**	**0.09**	**1.32**	**1.07**

Tab. S5: Calculation of the furnace averages with cooling rate correction of Korsimoro (KRS), Doumbala (DMB) and Siola (SIO) specimens. Ba is the specimen archaeointensity; CRC the cooling rate correction factor; Ba_{cr} is the cooling rate corrected intensity; Av_{samp} is the (cooling rate corrected) sample average; Av_{furn} is the (cooling rate corrected) furnace average and σ_{furn} is its corresponding standard deviation in μT and in %; and N_{meas}/N_{acc} is the ration between the number of specimens measured and accpeted. CRCs with a ° are averaged, whereas those without * are specimens correction factors. Specimens with a star were rejected because there was only one specimen available for the average. Number 1 to 40 were obtained with the Thellier-Coe protocol, number 41 to 90 are from Kapper et al.[1]. Numbers 91 to 121 were obtained with the pseudoThellier protocol. Pseudo-Thellier specimens marked with '2' were measured in Zurich.

	Specimen	Ba (μT)	CRC	Ba_{cr} (μT)	Av_{samp} (μT)	Av_{furn} (μT)	σ_{furn} (μT/%)	N_{meas}/N_{acc}
1	DMB1-12-07	33.8	0.981	33.2				
2	DMB1-12-08	32.3	0.927	29.9				
3	DMB1-12-09	32.5	0.951	30.9				
4	DMB1-12-10	31.4	0.941	29.6				
5	DMB1-12-12	32.6	0.941	30.7	30.8	**30.8**	**1.4/4.6**	6/5
6	DMB2-52-13	32.1	0.975°	31.3				
7	DMB2-52-14	32.4	0.980	31.8				
8	DMB2-52-15	31.9	0.970	30.9				
9	DMB2-52-16	32.2	0.975°	31.4				
10	DMB2-52-17	32.1	0.975°	31.3				
11	DMB2-52-18	32.1	0.975°	31.3	31.3	**31.3**	**0.3/0.8**	6/6
12	DMB3-24-19	38.6	0.937	36.2				
13	DMB3-24-20	38.5	0.954	36.7				
14	DMB3-24-21	37.4	0.940	35.2				
15	DMB3-24-22	38.8	0.944	36.6				
16	DMB3-24-23	38.7	0.955	37.0				
17	DMB3-24-24	39.8	0.954	38.0	36.6	**36.6**	**0.9/2.5**	6/6
18	KRS28-01-01	49.3	0.989	48.7				
19	KRS28-01-02	48.7	0.976°	47.5				
20	KRS28-01-03	43.4	0.988	42.9				
21	KRS28-01-04	47.4	0.987	46.8				

	Specimen	Ba (μT)	CRC	Ba$_{cr}$ (μT)	Av$_{samp}$ (μT)	Av$_{furn}$ (μT)	σ_{furn} (μT/%)	N_{meas}/N_{acc}
22	KRS28-01-05	52.1	0.940	49.0				
23	KRS28-01-06	54.0	0.976°	52.7	47.9	**47.9**	**3.2/6.7**	6/6
24	SIO1BA-27	45.9	0.939	43.1				
25	SIO1BA-28	51.4	0.884	45.4				
26	SIO1BA-29	38.5	0.912°	35.1	41.2	**41.2**	**5.4/13.2**	6/3
27	SIO4BA-37	43.8	0.975°	41.9				
28	SIO4BA-38	48.1	0.899	43.3				
29	SIO4BA-39	51.4	0.977	50.2				
30	SIO4BA-40	48.3	0.973	47.0				
31	SIO4BA-41	48.3	0.978	47.2				
32	SIO4BA-42	55.0	0.960	52.8	47.1	**47.1**	**4.1/8.7**	6/6
33	SIO5GA-44	43.5	0.983°	42.7				
34	SIO5GA-45	48.1	0.983°	47.3				
35	SIO5GA-46	45.4	0.983°	44.6				
36	SIO5GA-47	46.6	0.983°	45.8				
37	SIO5GA-48	44.8	0.983°	44.0	44.9			
38	SIO5GC-50	47.0	0.969	45.5				
39	SIO5GC-51	43.1	0.971	41.8				
40	SIO5GC-52	40.4	0.974	39.3	42.2	**43.6**	**1.9/4.3**	12/8
41	KRS05-04-J	53.0	0.950°	50.4				
42	KRS05-04-K	51.6	0.950°	49.0	49.7			
43	KRS05-08-K	53.4	0.950°	50.7				
44	KRS05-08-L	54.4	0.950°	51.7	51.2			
45	KRS05-09-I	53.7	0.950°	51.0				
46	KRS05-09-M	55.6	0.950°	52.8	51.9	**50.9**	**1.1/2.2**	16/6
47	KRS06-05-F	54.6	0.950°	51.9	51.9			
48	KRS06-07-K	38.7	0.950°	36.8				
49	KRS06-07-O	42.6	0.950°	40.5	38.6	**45.3**	**9.4/20.7**	3/3
50	KRS07-04-F*	16.8	0.950°	16.0				3/1

	Specimen	Ba (μT)	CRC	Ba_{cr} (μT)	Av_{samp} (μT)	Av_{furn} (μT)	σ_{furn} (μT/%)	N_{meas}/N_{acc}
51	KRS10-03-D	35.1	0.938	32.9				
52	KRS10-03-G	34.1	0.948°	32.3				
53	KRS10-03-H	33.9	0.948°	32.1	32.5			
54	KRS10-04-F	29.0	0.948°	27.5				
55	KRS10-04-G	31.1	0.948°	29.5	28.5	**30.5**	**2.8/9.2**	8/5
56	KRS13-06-E	53.8	0.950°	51.1				
57	KRS13-06-F	53.5	0.950°	50.8	51.0	**51.0**	**0.2/0.4**	3/2
58	KRS17-04-F*	39.3	0.950°	37.3				3/1
59	KRS21-09-A*	33.8	0.969°	32.7				6/1
60	KRS23-04-A	32.6	0.970°	31.6				
61	KRS23-04-C	36.0	0.970°	34.9				
62	KRS23-04-I	37.7	0.964	36.3	34.3			
63	KRS23-05-A	26.2	1.166	30.5				
64	KRS23-05-F	39.8	0.902	35.9	33.2			
65	KRS23-06-D	45.0	0.970°	43.7				
66	KRS23-06-I	44.0	0.970°	42.7	43.2			
67	KRS23-07-I	46.8	0.901	42.2	42.2	**38.2**	**5.2/13.6**	12/8
68	KRS24-05-V	45.6	0.939°	42.8				
69	KRS24-05-Y	45.3	0.939°	42.5	42.7	**42.7**	**0.2/0.5**	9/2
70	KRS30-08-C	45.8	0.950°	43.5				
71	KRS30-08-D	41.2	0.950°	39.1	41.3	**41.3**	**3.1/7.6**	3/2
72	KRS33-01-A	59.3	0.950°	56.3				
73	KRS33-01-B	68.2	0.950°	64.8				
74	KRS33-01-C	78.9	0.950°	75.0	65.4	**65.4**	**9.3/14.3**	3/3
75	KRS34-05-E	40.7	0.940°	38.3				
76	KRS34-05-F	38.6	0.940°	36.3				
77	KRS34-05-N	40.7	0.940°	38.3	37.6			
78	KRS34-06-D	40.8	0.940°	38.3				
79	KRS34-06-F	41.7	0.940°	39.2				

	Specimen	Ba (μT)	CRC	Ba_{cr} (μT)	Av_{samp} (μT)	Av_{furn} (μT)	σ_{furn} (μT/%)	N_{meas}/N_{acc}
80	KRS34-06-I	42.3	0.940°	39.8	39.1	**38.3**	**1.1/2.8**	12/6
81	KRS35-01-A	39.4	0.922°	36.3				
82	KRS35-01-D	34.7	0.922°	32.0				
83	KRS35-01-E	34.6	0.922°	31.9	33.4			
84	KRS35-05-A	36.9	0.922°	34.0				
85	KRS35-05-B	33.2	0.922°	30.6				
86	KRS35-05-F	34.9	0.972	33.9				
87	KRS35-05-I	35.3	0.922°	32.5	32.8			
88	KRS35-07-F	37.0	0.881	32.6	32.6	**32.9**	**0.4/1.3**	13/8
89	KRS3D-03-A*	36.2	0.952°	34.5				
90	KRS4B-01-A*	45.6	0.930°	42.4				
91	DMB1-1-3	27.2						
92	DMB1-1-5	35.2						
93	DMB1-1-6	34.3						
94	DMB1-1-7	34.2			32.7	**32.7**	**3.7/11.4**	5/4
95	DMB2-5-3	29.5						
96	DMB2-5-4	29.6						
97	DMB2-5-5	32.9						
98	DMB2-5-7	33.5			31.4	**31.4**	**2.1/6.8**	5/4
99	DMB6-6-3	38.6						
100	DMB6-6-7	36.4						
101	DMB6-6-8	39.6			38.2	**38.2**	**1.6/4.2**	5/3
102	KRS06-05-D*	47.8						5/1
103	KRS10-04-H*[2]	32.0						3/1
104	KRS21-02-H	22.6						
105	KRS21-09-C	29.6			26.1	**26.1**	**4.9/18.9**	4/2
106	KRS23-04-B[2]	37.6						
107	KRS23-04-C	23.9			30.7			
108	KRS23-05-C	31.9			31.9			

	Specimen	Ba (μT)	CRC	Ba$_{cr}$ (μT)	Av$_{samp}$ (μT)	Av$_{furn}$ (μT)	σ_{furn} (μT/%)	N_{meas}/N_{acc}
109	KRS23-06-A	38.6						
110	KRS23-06-G	32.9			35.7			
111	KRS23-07-J	30.9			30.9	**32.3**	**2.3/7.2**	7/6
112	KRS28-01-I*	33.8						5/1
113	KRS30-08-B	28.4						
114	KRS30-08-F	27.0						
115	KRS30-08-M^2	51.2			35.5	**35.5**	**13.6/38.3**	6/3
116	KRS35-05-H	20.6						
117	KRS35-08-D	24.0			22.3	**22.3**	**2.5/11.0**	5/2
118	SIO1A-1	28.3						
119	SIO1B-1	27.1			27.7	**27.7**	**0.8/3.0**	5/2
120	SIO3B-1*	44.2						4/1
121	SIO5A-1*	38.8						5/1

Tab. S6: Data compilation used for the reference curves. Age and σ_{Age} are the age and age uncertainty, respectively. Ba and σ_{Ba} are the intensity and its standard error, and Ba_{rl} (μT) is the relocated intensity. Lat and Lon are latitude and longitude. The different countries are indicated as follows: MA = Mali, SE = Senegal, LI = Libya, MO = Morocco, TU = Tunisia, BF = Burkina Faso, CI = Canary Islands, IC = Ivory Coast. GEOM in the 'Reference' column indicates that the data was obtained from the Geomagia50.v3.2[2] database. KRS, DMB and SIO are results from this study or from Kapper et al.[1] from Korsimoro, Doumbala and Siola, respectively. TT and PT indicate results from Thellier or pseudoThellier experiment.

Nr.	Age (yr AD)	σ_{Age} (yrs)	Ba (μT)	Ba_{rl} (μT)	σ_{Ba} (μT)	Lat (°)	Lon (°)	Country	Reference
1	27.0	47.0	40.1	43.6	2.5	13.71	-4.51	MA	3, GEOM
2	124.0	73.0	38.9	41.3	3.1	16.01	-13.61	SE	4, GEOM
3	125.0	25.0	55.0	47.2	0.0	32.80	13.10	LI	3, GEOM
4	125.0	48.0	41.9	45.6	0.4	13.71	-4.51	MA	5, GEOM
5	150.0	50.0	53.3	44.2	4.2	35.50	-6.00	MO	6
6	150.0	150.0	86.6	72.0	14.1	35.38	11.03	TU	6
7	200.0	100.0	71.0	58.8	8.8	35.67	10.87	TU	3, GEOM
8	221.0	70.0	36.2	38.4	1.6	16.01	-13.61	SE	5, GEOM
9	250.0	50.0	58.1	49.0	4.8	34.10	-5.50	MO	7
10	262.0	160.0	60.4	50.2	5.3	35.26	11.00	TU	3, GEOM
11	271.0	58.0	35.9	38.4	1.5	15.31	-5.51	MA	3
12	315.5	94.5	34.9	37.1	1.2	16.01	-13.61	SE	6
13	436.5	83.5	95.5	78.5	15.1	36.25	10.40	TU	5, GEOM
14	450.0	50.0	54.5	46.0	4.1	34.10	-5.50	MO	8
15	450.0	50.0	63.9	52.4	10.6	36.44	10.72	TU	3, GEOM
16	485.0	75.0	36.4	38.7	2.7	16.01	-13.61	SE	3, GEOM
17	502.0	72.0	35.3	37.6	3.3	15.81	-13.31	SE	8
18	550.0	50.0	37.5	30.8	8.0	36.48	10.78	TU	3
19	563.0	61.0	38.8	41.2	1.3	15.81	-13.31	SE	3, GEOM
20	624.0	52.0	42.2	45.0	1.5	15.81	-13.31	SE	3, GEOM
21	664.0	31.0	43.4	47.2	3.5	13.71	-4.51	MA	KRS33, TT
22	720.0	60.0	65.4	71.8	9.3	12.81	-0.99	BF	KRS05, TT

Nr.	Age (yr AD)	σ_{Age} (yrs)	Ba (μT)	Ba$_{rl}$ (μT)	σ_{Ba} (μT)	Lat (°)	Lon (°)	Country	Reference
23	800.0	90.0	50.9	56.0	1.1	12.81	-1.06	BF	KRS06, TT
24	800.0	90.0	45.3	49.7	9.4	12.81	-1.06	BF	3, GEOM
25	815.0	81.0	37.0	39.3	4.1	15.81	-13.31	SE	9, GEOM
26	852.0	52.0	65.2	54.0	6.2	35.60	10.06	TU	8
27	863.0	88.0	51.4	42.5	5.9	35.78	10.83	TU	3, GEOM
28	899.0	56.0	39.6	42.4	1.0	15.31	-5.51	MA	10
29	925.0	50.0	43.6	39.6	4.4	28.51	-17.83	CI	9, GEOM
30	1028.0	28.0	62.8	52.0	12.7	35.66	10.10	TU	9, GEOM
31	1028.0	28.0	62.7	51.9	5.7	35.66	10.10	TU	9, GEOM
32	1028.0	28.0	58.0	48.0	4.5	35.66	10.10	TU	9, GEOM
33	1028.0	28.0	58.6	48.5	4.3	35.66	10.10	TU	11
34	1055.0	155.0	57.9	52.7	6.4	28.27	-16.73	CI	12
35	1058.0	0.0	81.6	74.3	0.1	28.27	-16.63	CI	KRS35, TT
36	1100.0	75.0	32.9	36.2	0.4	12.81	-1.06	BF	KRS35, PT
37	1100.0	75.0	22.3	24.5	2.5	12.81	-1.06	BF	KRS24, TT
38	1120.0	90.0	42.7	46.9	0.2	12.79	-1.09	BF	8
39	1200.0	25.0	44.1	36.6	2.5	35.51	11.08	TU	8
40	1200.0	25.0	46.1	38.3	4.7	35.51	11.08	TU	8
41	1200.0	25.0	48.6	40.3	1.6	35.51	11.08	TU	8
42	1200.0	25.0	46.6	38.7	3.7	35.51	11.08	TU	KRS34, TT
43	1290.0	20.0	38.3	42.1	1.1	12.81	-1.06	BF	KRS10, TT
44	1340.0	55.0	30.5	33.5	2.8	12.79	-1.09	BF	9, GEOM
45	1350.0	60.0	45.7	38.5	4.4	34.31	-5.93	MO	DMB6, PT
46	1350.0	65.0	38.2	43.1	1.6	9.88	-7.41	IC	KRS21, PT
47	1400.0	100.0	26.1	28.7	4.9	12.79	-1.09	BF	KRS23, TT
48	1430.0	20.0	38.2	42.0	5.2	12.79	-1.09	BF	KRS23, PT
49	1430.0	20.0	32.3	35.5	2.3	12.79	-1.09	BF	13
50	1470.0	0.0	41.5	37.9	7.4	28.00	-15.60	CI	10
51	1481.0	0.0	36.0	32.6	11.3	28.65	-17.86	CI	SIO4, TT
52	1545.0	55.0	47.1	53.1	4.1	9.86	-7.45	IC	11

Nr.	Age (yr AD)	σ_{Age} (yrs)	Ba (μT)	Ba_{rl} (μT)	σ_{Ba} (μT)	Lat (°)	Lon (°)	Country	Reference
53	1550.0	110.0	71.5	65.1	16.5	28.28	-16.77	CI	DMB3, TT
54	1550.0	100.0	36.6	41.3	0.9	9.88	-7.41	IC	10
55	1585.0	0.0	46.4	42.1	18.0	28.58	-17.89	CI	14, GEOM
56	1595.0	7.0	42.2	37.1	3.6	31.00	-8.00	MO	10
57	1646.0	0.0	44.2	40.1	0.4	28.52	-17.82	CI	KRS13, TT
58	1650.0	150.0	51.0	56.0	0.2	12.81	-1.06	BF	KRS28, TT
59	1650.0	50.0	47.9	52.7	3.2	12.79	-1.09	BF	KRS30, TT
60	1650.0	50.0	41.3	45.4	3.1	12.79	-1.09	BF	KRS30, PT
61	1650.0	50.0	35.5	39.0	13.6	12.79	-1.09	BF	10
62	1677.0	0.0	46.6	42.3	0.0	28.48	-17.87	CI	13
63	1704.0	0.0	43.1	39.2	4.9	28.25	-16.60	CI	13
64	1705.0	0.0	37.3	34.0	2.1	28.25	-16.60	CI	12
65	1706.0	0.0	39.4	35.9	0.9	28.27	-16.63	CI	11
66	1706.0	0.0	49.9	45.4	3.9	28.36	-16.76	CI	10
67	1712.0	0.0	45.4	41.2	2.8	28.55	-17.87	CI	15
68	1733.5	0.0	40.2	36.2	1.7	29.01	-13.76	CI	SIO1B, TT
69	1815.0	135.0	41.2	46.5	5.4	9.86	-7.45	IC	SIO1B, PT
70	1815.0	135.0	27.7	31.3	0.8	9.86	-7.45	IC	15
71	1824.0	0.0	41.4	37.3	2.3	29.01	-13.75	CI	SIO5, TT
72	1870.0	60.0	43.6	49.2	1.9	9.86	-7.45	IC	DMB1, TT
73	1895.0	80.0	30.8	34.8	1.4	9.88	-7.41	IC	DMB1, PT
74	1895.0	80.0	32.7	36.9	3.7	9.88	-7.41	IC	DMB2, TT
75	1895.0	80.0	31.3	35.3	0.3	9.88	-7.41	IC	DMB2, PT
76	1895.0	80.0	31.4	35.4	2.1	9.88	-7.41	IC	13
77	1909.0	0.0	42.6	38.8	4.0	28.25	-16.60	CI	10
78	1949.0	0.0	34.7	31.4	8.1	28.59	-17.88	CI	16
79	1949.0	0.0	40.5	36.6	0.0	28.89	-17.87	CI	10
80	1971.0	0.0	35.8	32.5	7.9	28.46	-17.85	CI	10

References

1. Kapper, L. et al. Reconstructing the Geomagnetic Field in West Africa: First Absolute Intensity Results from Burkina Faso. Sci. Rep. 7, 12 (2017).

2. Brown, M. C. et al. GEOMAGIA50.v3: 1. general structure and modifications to the archeological and volcanic database. Earth, Planets Sp. 67, 67–83 (2015).

3. Mitra, R., Tauxe, L., Keech McIntoshf, S. Two thousand years of archeointensity from West Africa. Earth Planet. Sci. Lett. 364, 123–133 (2013).

4. Odah, H. Using the Thellier Method. J. Geomagn. Geoelectr. 47, 41–58 (1995).

5. Kovacheva, M. A. Some Archaeomagnetic Conclusions from 3 Archaeological Localities in Northwest Africa. Dokladi na Bolgarskata Akademiya na Naukite 37(2), 171-174 (1984).

6. Fouzai, B., Casas, L., Laridhi Ouazaa, N., Fantar, M., Álvarez, A. Archaeomagnetic data from three Roman sites in Tunisia. J. Archaeol. Sci. 40, 1703–1714 (2012).

7. Fouzai, B., Casas, L., Laridhi Ouazaa, N., Fantar, M., Álvarez, A. Archaeomagnetic data from three Punic sites in Tunisia. J. Archaeol. Sci. 40, 1703–1714 (2013).

8. Casas, L. et al. New Archaeomagnetic Data from Tunisia: Dating of Two Kilns and New Archaeointensities from Three Ceramic Artifacts. Geoarchaeology 31, 564–576 (2016).

9. Gómez-Paccard, M. et al. Archaeomagnetic and rock magnetic study of six kilns from North Africa (Tunisia and Morocco). Geophys. J. Int. 189, 169–186 (2012).

10. Monster, M. W. L., de Groot, L. V., BIGGIN, A. J., Dekkers, M. J. The performance of various palaeointensity techniques as a function of rock magnetic behaviour – A case study for La Palma. Phys. 242, 36–49 (2015).

11. Kissel, C. et al. Holocene geomagnetic field intensity variations: Contribution from the low latitude Canary Islands site. Earth Planet. Sci. Lett. 430, 178–190 (2015).

12. de Groot, L. V. et al. High paleointensities for the Canary Islands constrain the Levant geomagnetic high. Earth Planet. Sci. Lett. 419, 154–167 (2015).

13. Sherwood, G. J. Evaluation of a multi-specimen approach to palaeointensity determination. J. Geomagn. Geoelectr. 43, 341–349 (1991).

14. Casas, L., Briansó, J. L., Álvarez, A., Benzzi, K., Shaw, J. Archaeomagnetic intensity data from the Saadien Tombs (Marrakech, Morocco), late 16th century. Phys. Chem. Earth 33, 474–480 (2008).

15. Calvo-Rathert, M., Morales-Contreras, J., Carrancho, Á., Goguitchaichvili, A. A comparison of Thellier-type and multispecimen paleoinetnsity determinations on Pleistocene and historical lava flows from Lanzarote (Canary Islands, Spain). Geochemistry Geophys. Geosystems 17, 2825–2834 (2016).

16. Valet, J. P., Herrero-Bervera, E., Carlut, J., Kondopoulou, D. A selective procedure for absolute paleointensity in lava flows. Geophys. Res. Lett. 37, 1–7 (2010).
